# Genetic imputation of transcriptome and proteome illuminates novel therapeutic targets of cutaneous melanoma

**DOI:** 10.1093/bib/bbaf564

**Published:** 2025-10-28

**Authors:** Yantao Xu, Poyee Lau, Jing Wang, Xiao-Rui Qiu, Zixi Jiang, Danyang Liu, Shuang Zhao, Lin Zhu, Xiang Chen, Weichu Sun, Jia-Chen Liu

**Affiliations:** Department of Dermatology, The Third Affiliated Hospital, Sun Yat-sen University, Tianhe District, Guangzhou 510630, Guangdong, China; Department of Dermatology, Shenzhen People's Hospital (The First Affiliated Hospital, Southern University of Science and Technology; The Second Clinical Medical College, Jinan University), Luohu District, Shenzhen 518020, Guangdong, China; Xiangya School of Medicine, Central South University, Yuelu District, Changsha 410013, Hunan, China; Xiangya School of Medicine, Central South University, Yuelu District, Changsha 410013, Hunan, China; Xiangya Hospital, Central South University, Kaifu District, Changsha 410008, Hunan, China; Department of Dermatology, The Third Affiliated Hospital, Sun Yat-sen University, Tianhe District, Guangzhou 510630, Guangdong, China; Department of Dermatology, The First Affiliated Hospital, Sun Yat-sen University, Yuexiu District, Guangzhou 510080, Guangdong, China; The Center of Systems Biology and Data Science, Xiangya School of Medicine, Central South University, Yuelu District, Changsha 410013, Hunan, China; Department of Dermatology, Xiangya Hospital, Central South University, Kaifu District, Changsha 410008, Hunan, China; Department of Dermatology, Xiangya Hospital, Central South University, Kaifu District, Changsha 410008, Hunan, China; Molecular Biology Research Center, Center for Medical Genetics, Hunan Province Key Laboratory of Basic and Applied Hematology, School of Life Sciences, Central South University, Yuelu District, Changsha 410013, Hunan, China; Department of Dermatology, Xiangya Hospital, Central South University, Kaifu District, Changsha 410008, Hunan, China; Xiangya Hospital, Central South University, Kaifu District, Changsha 410008, Hunan, China; Department of Spine Surgery, The Second Xiangya Hospital, Central South University, Furong District, Changsha 410011, Hunan, China; Xiangya School of Medicine, Central South University, Yuelu District, Changsha 410013, Hunan, China; Xiangya Hospital, Central South University, Kaifu District, Changsha 410008, Hunan, China; The Center of Systems Biology and Data Science, Xiangya School of Medicine, Central South University, Yuelu District, Changsha 410013, Hunan, China

**Keywords:** genome-wide association studies, cutaneous melanoma, drug repositioning, protein, biomarker, therapeutic target

## Abstract

Genomic heterogeneity in melanoma tumors remains a major obstacle to achieving durable responses with conventional and targeted therapies. In this study, we performed a genome-wide association study meta-analysis, integrated with proteome-wide Mendelian randomization and colocalization analyses, to identify potential therapeutic targets for cutaneous melanoma (CM). Analyzing data from 5527 CM cases and 645 797 controls, we uncovered seven novel genome-wide significant variants linked to CM risk. Additionally, genetically predicted protein levels revealed 15 proteins associated with CM susceptibility, among which ASIP, CD72, CCL11, LYZ, and CCL25 showed the strongest associations. Validation in independent cohorts further supported their potential as biomarkers. Notably, these protein-coding genes are predominantly expressed in macrophages, B cells, CD8 T cells, and malignant cells within CM tissue. Among them, CD72 and LYZ stand out as promising candidates for therapeutic repurposing. These findings enhance our understanding of CM-related genetic and protein biomarkers, providing a foundation for future therapeutic development.

## Introduction

Cutaneous melanoma (CM) is a highly aggressive skin cancer, accounting for less than 5% of all cutaneous malignancies but responsible for the majority of skin cancer-related deaths [1]. In 2020 alone, over 300 000 new CM cases were reported worldwide, with ~57 000 deaths attributed to the disease [2]. Over the past two decades, CM incidence has steadily increased, underscoring the urgent need for improved early detection and effective therapeutic strategies [3, 4]. While immune checkpoint blockade (ICB) therapies have significantly improved survival rates, identifying reliable biomarkers remains essential for optimizing early diagnosis and treatment response [5, 6]. Despite advancements in understanding CM risk factors [7], translating this knowledge into effective preventive interventions remains a challenge. Non-invasive biomarkers for early diagnosis and therapeutic monitoring are critically needed.

Genomic studies provide valuable insights into complex diseases like CM by minimizing confounding biases and reverse causation [8]. Large-scale genome-wide association studies (GWAS) have successfully mapped genetic variants associated with CM risk. A recent GWAS including 36 760 CM cases and 375 188 controls identified 54 CM-associated loci [9]. These discoveries offer promising avenues for understanding CM pathogenesis and developing novel preventive strategies.

Blood-based biomarkers play a central role in clinical diagnostics and treatment decision-making [10]. Circulating protein profiles offer a promising approach for early disease detection and risk assessment [11]. Several studies have explored associations between circulating proteins and CM risk [12–16]; however, these studies often suffer from limited protein coverage, small sample sizes, and an inability to establish causality.

Recent advancements in proteomic technologies now allow for the simultaneous measurement of thousands of circulating proteins from a single blood sample [17, 18], facilitating the integration of genomic and proteomic data for biomarker discovery. Combining GWAS data with protein quantitative trait loci (pQTL) can provide deeper mechanistic insights into CM development and help identify novel biomarkers and therapeutic targets.

In this study, we conducted a GWAS meta-analysis using publicly available CM datasets to identify genetic variants associated with CM risk. We integrated proteome-wide Mendelian randomization (MR) with pQTL data from the Icelandic cohort to assess causal relationships between circulating proteins and CM susceptibility. To enhance biomarker credibility, we performed two-sample MR and genetic colocalization analyses, followed by validation in independent CM cohorts, including those receiving ICB therapy. Additionally, single-cell expression analysis was conducted to determine the cellular sources of key proteins within CM biopsy datasets. Further functional analyses, including CM risk factor assessment, molecular docking, druggability evaluation, and transcriptomic analysis, were carried out to explore the biological significance of our findings.

## Method

### CM population and datasets

**Figure 1:**
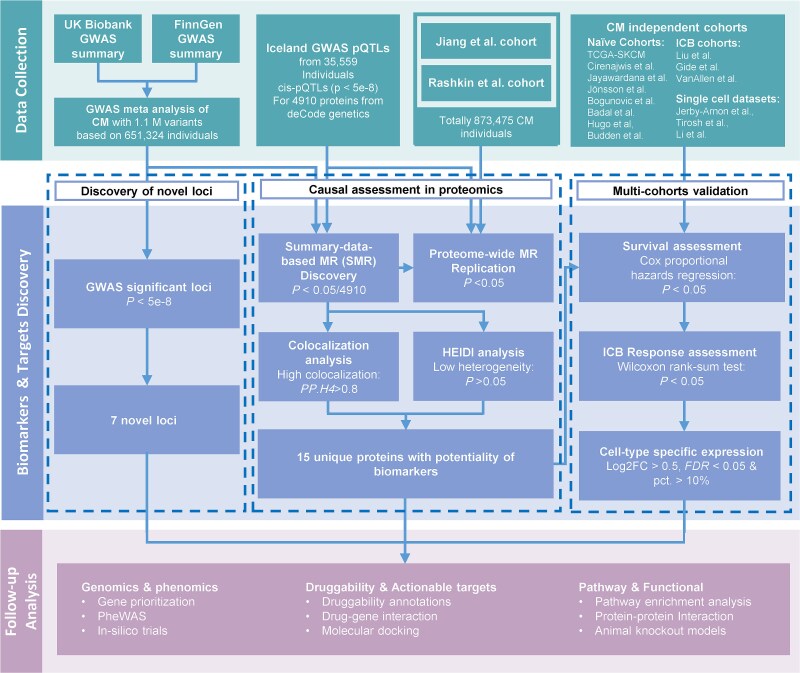
Flowchart of the study design.

An overview of the study is shown in Fig. 1. This study utilized GWAS data from two European-ancestry cohorts. Cohort 1 included 2534 CM cases identified based on the ICD-10 code C43.9 and 358 660 controls from the UK Biobank (UKB) [19]. Cohort 2 consisted of 2993 CM cases, diagnosed through clinical evaluation, ICD codes, and imaging, along with 287 137 controls from FinnGen (R10) [20]. Additional GWAS summary datasets were incorporated for replication, including Jiang et al. (2824 cases, 453 524 controls) [21], Rashkin et al. (6777 cases, 410 350 controls) [22], and Loh et al. (skin pigmentation GWAS with 548 203 individuals) [23] (Supplementary Table S1). Further details on genotyping, imputation, and quality control are provided in the supplementary information. Genotype imputation in the UK Biobank was conducted using the Haplotype Reference Consortium (HRC) and UK10K reference panels, while FinnGen used the population-specific SISu v3 panel. To mitigate potential biases arising from differences in reference panels, we applied stringent quality control filters by retaining only variants with a minor allele frequency (MAF) > 0.005 and an imputation INFO score > 0.9 in both datasets.

### GWAS meta-analysis for CM

A fixed-effects inverse-variance weighted (IVW) meta-analysis was conducted using GWAMA [24] to identify genetic variants associated with CM. A genome-wide significance threshold of *P* < 5 × 10^−8^ was applied, and variants with a minor allele frequency (MAF) < 0.5% were excluded, yielding 1 107 727 associations. Linkage disequilibrium (LD) was assessed using a reference set of 5000 randomly selected UKB European-ancestry individuals (LDEUR).

### Gene-level and pathway enrichment analysis

Genetic variants were functionally annotated using FUMA [25] to identify independent genomic loci. MAGMA [26] was used for gene-based association testing and pathway enrichment analysis across Gene Ontology, KEGG, and Reactome databases. Pathways with an FDR < 0.05 were considered significantly enriched.

### Polygenic priority score for gene prioritization

Gene prioritization was performed using polygenic priority score (PoPS), an integrative framework that incorporates GWAS summary statistics with gene expression, pathway data, and protein–protein interaction networks. MAGMA-derived gene association statistics were used to assess enrichment across biological features. A joint enrichment analysis was conducted using a leave-one-chromosome-out (LOCO) framework, and PoPS scores were calculated for 18 076 genes. Candidate genes within a 500 kb window of GWAS loci were selected based on the highest PoPS score.

### Polygenic priority score

To prioritize candidate genes associated with CM, we applied PoPS, a computational framework that integrates GWAS summary statistics with diverse biological data sources, including gene expression profiles, pathway annotations, and protein–protein interaction networks. PoPS has been demonstrated to outperform traditional similarity-based or locus-based prioritization methods, particularly in identifying causal genes at non-coding GWAS loci.

The PoPS analysis was conducted in multiple steps. First, MAGMA was used to compute gene-level association statistics, generating z-scores and gene–gene correlation matrices based on GWAS summary statistics and LD information from the 1000 Genomes Project. Next, PoPS performed marginal feature selection, independently assessing the enrichment of each gene feature using generalized least squares modeling. Only features meeting the predefined nominal significance threshold (*P* < .05) were retained for further analysis.

In the subsequent phase, PoPS executed joint enrichment analysis, incorporating all selected gene features simultaneously within a LOCO framework. This approach ensured that gene prioritization remained unbiased by excluding each gene’s corresponding chromosome during the modeling process. A broad range of biological features—including chromatin accessibility data, epigenomic markers, and tissue-specific expression profiles—were considered to improve the prioritization accuracy. The complete list of gene features utilized in PoPS is publicly available at https://github.com/FinucaneLab/gene_features.

Finally, PoPS computed polygenic priority scores for 18 076 genes based on the joint model incorporating all selected gene features. Each PoPS score was assigned independently of the GWAS data on the chromosome where the gene was located, ensuring robustness against confounding effects from local genetic variation. For downstream analyses, genes within a 500 kb genomic window surrounding significant GWAS loci were annotated using Ensembl gene models, and the gene with the highest PoPS score within each locus was designated as the most likely candidate.

Although PoPS provides a comprehensive framework for gene prioritization, it is important to acknowledge its limitations. The model relies on existing gene expression datasets, protein interaction networks, and pathway annotations, which may not fully capture functional variants acting through mechanisms beyond the current scope of biological databases. Nevertheless, PoPS has proven effective in identifying putative causal genes for complex traits, making it a valuable tool for uncovering novel therapeutic targets in CM.

### Proteome-wide MR and summary-based MR

pQTL data were derived from a proteomic study of 4907 aptamers measured in 35 559 Icelandic individuals [27]. Genetic instruments were selected based on genome-wide significance (*P* < 5 × 10^−8^), and variants in the MHC region (chr6:25.5–34.0 Mb) were excluded due to complex LD. Independent pQTLs were identified through LD clumping (r^2^ < 0.001). Summary-data-based Mendelian randomization (SMR) analysis was conducted using SMR software (Linux v1.0.3) to assess pleiotropic associations between genetically determined protein levels and CM risk [28].

### Proteome-wide MR analysis

To assess the causal relationship between circulating protein levels and CM risk, we conducted bidirectional two-sample MR, examining both forward and reverse directions. Forward MR tested whether genetically predicted protein levels influence CM susceptibility, while reverse MR assessed whether CM risk variants affect circulating protein levels, helping to clarify potential reverse causation.

Genetic instruments for circulating proteins were derived from a genome-wide proteomic study in 35 559 Icelandic individuals, selecting SNPs strongly associated with protein levels at *P* < 5 × 10^−8^. LD clumping (r^2^ < 0.001) ensured instrument independence, and SNPs in the MHC region were excluded due to complex linkage structures. Instrument strength was evaluated using the F-statistic, with F > 10 considered sufficient to minimize weak instrument bias. For reverse MR, independent CM-associated variants (*P* < 5 × 10^−8^) were selected from our GWAS meta-analysis to test their influence on protein levels.

Causal relationships between protein levels and CM were evaluated using TwoSampleMR [29] and MR-PRESSO [30]. Causal estimates were primarily obtained using IVW [31] regression, which provides the most precise effect estimates under the assumption of valid instruments. Sensitivity analyses included MR-Egger regression to detect and correct for pleiotropy, the weighted median estimator for robustness when up to 50% of instruments are invalid, and MR-RAPS [32] to mitigate weak instrument bias. Maximum likelihood estimation [33] was also applied to account for uncertainty in gene-protein and gene-CM associations. To assess heterogeneity, Cochran’s Q [34] test was performed, while leave-one-out analyses examined the influence of individual SNPs. MR-Egger [35] and MR-PRESSO [30] was used to detect and remove outliers when pleiotropy was evident.

In forward MR, a significant association between higher protein levels and increased CM risk suggests a potential oncogenic role, whereas an inverse association may indicate a protective effect. If reverse MR does not support a causal effect of CM on protein levels, this strengthens the evidence for a unidirectional causal link from proteins to CM rather than disease-driven proteomic changes. However, despite robust statistical approaches, residual pleiotropy remains a potential concern, and effect estimates are constrained by the proportion of protein variance explained by genetic instruments. Additionally, as analyses were conducted in European-ancestry populations, findings may not be directly generalizable to other ethnic groups.

### Colocalization analyses

Colocalization between GWAS and pQTL associations was assessed using the heterogeneity in dependent instruments (HEIDI) test and Bayesian colocalization analysis via the coloc R package (v5.1.0) [36]. SNPs within ±100 kb of the lead variant were analyzed, with a posterior probability of shared causal variants (posterior probability of H4 [PP.H4] > 0.8) considered strong evidence of colocalization.

### Association with CM risk factors and safety traits

Genetic variants and SMR-identified proteins were assessed for associations with nine CM risk factors and nine safety traits using European-ancestry GWAS data [19, 23, 37–42]. Directionality checks were performed to ensure biological consistency between risk factor associations and CM susceptibility.

### Querying the MGI database

To investigate the functional relevance of candidate genes identified through GWAS and MR/colocalization analyses, we queried the Mouse Genome Informatics (MGI) database (http://www.informatics.jax.org/). MGI provides a comprehensive repository of genetic, genomic, and phenotypic data from murine models, making it a valuable resource for assessing potential biological roles of CM-associated genes.

We examined all genes mapped to GWAS loci and those prioritized as causal through MR and colocalization analyses. MGI standardizes gene nomenclature and employs controlled vocabularies, including the Mouse Developmental Anatomy Ontology, Mammalian Phenotype Ontology, and Gene Ontologies, to systematically categorize gene functions and associated phenotypes. Data extraction focused on identifying system-wide abnormalities linked to queried genes, particularly phenotypes relevant to melanoma progression, immune regulation, and tumor suppression.

By integrating cross-species genetic evidence, we aimed to strengthen the biological plausibility of our findings, providing additional support for candidate genes as potential therapeutic targets.

### Druggability and functional evaluation

Proteins identified through GWAS and SMR analyses were classified based on drug tractability using OpenTargets [43]. Non-druggable proteins were cross-referenced with the Drug Signatures Database [44] to identify potential therapeutic targets.

### Validation in melanoma transcriptomic and single-cell datasets

Survival and treatment response validation was performed using TCGA-SKCM data, along with eight treatment-naive and seven ICB therapy cohorts from the Gene Expression Omnibus (GEO), the Sequence Read Archive (SRA), and the database of Genotypes and Phenotypes (dbGaP). (Supplementary Table S1). Single-cell RNA sequencing data from GSE115978, GSE72056, and GSE189889 were integrated using Harmony and analyzed using Seurat (v4.0.4). Differential expression analysis was performed using the Wilcoxon rank-sum test, with FDR < 0.05, log2FC > 0.5, and expression proportion > 10% considered as evidence of cell-type enrichment.

### Pathway and molecular docking analysis

Protein–protein interaction (PPI) networks were constructed using STRING, and pathway enrichment analysis was conducted using ReactomePA and clusterProfiler. Molecular docking was employed to assess the binding affinities of CD72 and LYZ to potential targets using AlphaFold structures [45] and HADDOCK docking software. Results were visualized using UCSF Chimera [46]. To further explore the druggability of CD72 and identify potential pharmacological activators, we performed molecular docking against a curated library of 1609 FDA-approved small-molecule compounds retrieved from the ZINC database. Binding affinities were calculated in kcal/mol, and compounds were ranked accordingly. All docking runs were performed in triplicate to ensure reproducibility.

### Molecular dynamics simulations

Molecular dynamics simulations were conducted to further evaluate the stability and dynamic behavior of the CD72–dihydroergotamine complex identified by molecular docking. All simulations were performed using GROMACS 2025 with the CHARMM36m force field. Each protein–ligand complex was solvated in a cubic box of TIP3P water molecules with a minimum distance of 1.0 nm from the protein surface, and the system was neutralized with counterions. Energy minimization was carried out using the steepest descent algorithm until the maximum force was reduced below 1000 kJ/mol/nm. The systems were subsequently equilibrated under NVT and NPT ensembles for 100 ps each, maintaining 300 K and 1 bar with the V-rescale thermostat and the Parrinello–Rahman barostat, respectively.

Production simulations were performed for 20 ns with an integration time step of 2 fs. Structural stability and dynamic properties were evaluated by calculating the root mean square deviation, root mean square fluctuation, and solvent-accessible surface area, using standard GROMACS utilities. Additionally, Ramachandran plots were generated to assess backbone dihedral angle distributions and stereochemical quality.

## Result

### Genome-wide meta-analysis identifies novel loci for cutaneous melanoma

**Figure 2:**
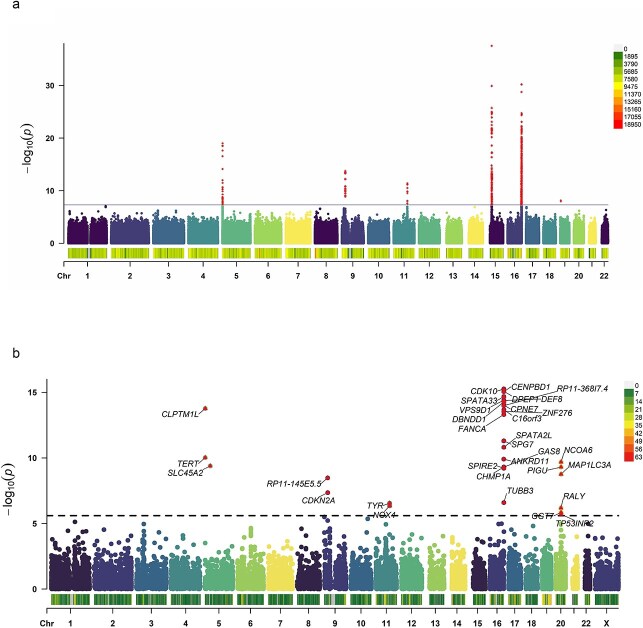
Manhattan plots showing associations with CM from GWAS meta-analysis (a) Manhattan plot showing the −log10(P value) of association for each SNP from the GWAS meta-analysis plotted on the y-axis against genomic position on the x-axis. The dotted horizontal line indicates the genome-wide significance threshold (*P* < 5 × 10^−8^). (b) Manhattan plot showing the −log10(P value) of association for each gene plotted against genomic position on the x-axis. The dotted horizontal line represents the Bonferroni-corrected significance threshold (P < 0.05/19,744). All tests were two-sided and adjusted for multiple comparisons.

The meta-analysis of CM was conducted using an inverse-variance weighted fixed-effects approach, integrating UKB data with publicly available GWAS summary statistics (Fig. 2, Supplementary Table S1). After quality control, association results for 11 078 728 genetic variants were obtained. The quantile-quantile plot demonstrated no substantial inflation of test statistics (Supplementary Fig. S1). A total of 111 genome-wide significant loci (*P* < 5 × 10^−8^) were identified, including seven novel variants located >1000 kb away from previously reported variants (Supplementary Table S2, Supplementary Fig. S2, Fig. 2a, Table 1). For each locus, we identified the nearest annotated feature, summarized its known or putative biological function, and proposed a hypothesis for its potential role in melanoma pathogenesis. (Supplementary Table S3) To validate these findings, we compared them against previously reported CM-associated SNPs, summarized in Supplementary Table S4. Most of the identified variants showed associations with at least one CM risk factor (Supplementary Table S5). Notably, rs1080476/CDH12 was linked to Ease of skin tanning, Facial ageing, Childhood sunburn occasions, Smoking status, and Job involves heavy manual work. rs78874486/MTAP and rs1873456/RNF216P1 were associated with multiple factors, including Childhood sunburn occasions and Income level, while rs77987651/DPEP1 showed links to educational attainment and Coffee intake.

**Table 1:** Novel loci reported for CM in the GWAS meta-analysis.

rsID	CHR	BP	nearestGene	POPs	CADD Phred score	EA	NEA	MAF	BETA	SE	P-value
rs41286740	1	67 956 176	SNORD65	MTAP	5.685	G	T	0.005681	0.07249	0.01059	7.80E-12
rs142267292	1	85 455 316	CADM2	MTAP	0.539	G	A	0.012124	0.04844	0.00711	9.74E-12
rs1873456	10	69 949 618	COL13A1	RNF216P1	4.277	A	G	0.409356	−0.00573	8.40E-04	1.05E-11
rs78874486	12	55 666 907	RP11-310H4.3	MTAP	1.756	T	C	0.008479	0.05934	0.00838	1.47E-12
rs1080476	15	49 988 854	ATP8B4	CDH12	5.204	A	G	0.278925	0.0123	0.00167	1.84E-13
rs78598578	3	65 305 132	RP11-766 N7.3	EPB41L1	7.787	T	C	0.010488	0.0565	0.00759	1.00E-13
rs77987651	6	94 424 839	EXOC6	DPEP1	2.606	G	A	0.010281	0.05087	0.00774	5.02E-11

Gene-based association analyses using MAGMA identified additional significant genes associated with CM (Fig. 2b, Supplementary Table S6). Given prior findings that PoPS outperforms other gene prioritization methods, we assigned priority to the nearest gene to each indexed SNP and the gene with the highest PoPS score within a 500 kb region. This complementary approach allowed for a more comprehensive functional annotation of CM-associated variants (Table 1). Fine-mapped genes and PoPS-prioritized genes were further assessed through gene-burden tests using putative loss-of-function (pLoF) variants from the Genebass-UK Biobank database.

Pathway enrichment analysis of CM-associated genes identified significant enrichment (FDR < 5%) in multiple biological processes, including melanin biosynthesis (adjusted *P* < .001), interferon signaling (*P* = .001), mitochondrial autophagy (*P* = .001), T helper 17 cell differentiation (*P* = .001), and response to interleukin-4 (*P* = .002) (Supplementary Table S7).

### SMR analysis identifies 15 causal protein biomarkers for CM

**Figure 3:**
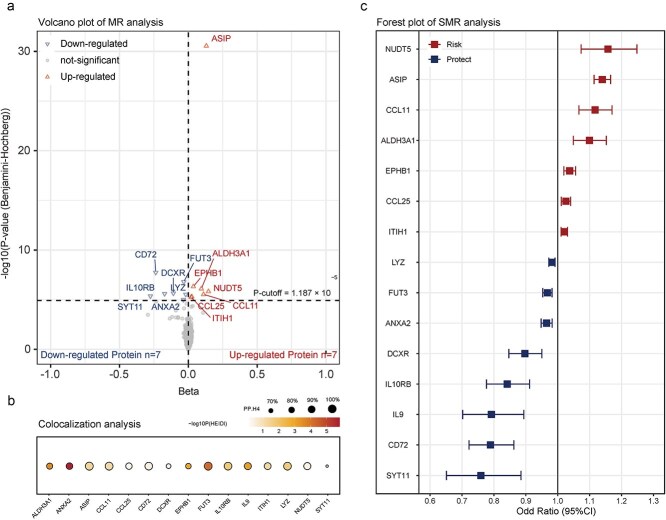
Result summary of SMR and colocalization analysis on the associations between plasma proteins and the risk of CM. (a, b) Volcano plot (a) and Forest plot (b) showing the Beta and P value from proteome wide SMR in the discovery stage. (c) Bubble chart exhibits the colocalization result of PP.H4 and P *HEIDI* of 15 proteins.

To establish functional links between genetic variants and CM, we performed SMR analysis integrating *cis-*pQTL data from the Icelandic Cancer Project and deCODE genetics. To minimize confounding, we applied a Bonferroni-corrected threshold (P_SMR < 1.187 × 10^−5^, *P* < .05/4212, Fig. 3a, b) and confirmed colocalization using Bayesian colocalization analysis (PP.H4 > 0.6, Fig. 3c, Supplementary Table S8). The HEIDI test (P_HEIDI >0.01) ruled out pleiotropic effects. External replication was conducted using two-sample MR, confirming associations for six proteins (Supplementary Table S9).

Based on replication and robustness of evidence, the 15 identified proteins were categorized into three tiers: (i) Tier 1 (high-confidence causal markers): ASIP, CD72, CCL11, LYZ, and CCL25, which passed all validation tests; (ii) Tier 2 (moderate confidence): FUT3, which failed colocalization or HEIDI analysis due to data limitations; (iii) Tier 3 (low confidence): EPHB1, ALDH3A1, NUDT5, DCXR, IL10RB, SYT11, ITIH1, ANXA2, and IL9, which failed MR replication or meta-analysis (Table 2).

**Table 2:** Summary results from SMR, MR, colocalization for 15 proteome-wide SMR-identified proteins.

Protein	Full name	SMR (discovery)		MR (replication)		Colocalization	P_HEIDI	Category
		β	P	β	P			
ASIP	Agouti Signaling Protein	1.31E-01	2.82E-31	5.68E-03	9.15E-05	100.00%	4.32E-02	tier 1
CD72	Cluster of Differentiation 72	−2.37E-01	1.81E-08	−4.87E-03	9.74E-03	97.40%	3.52E-01	tier 1
FUT3	Fucosyltransferase 3	−3.30E-02	1.60E-07	−3.86E-05	2.00E-02	99.20%	5.76E-05	tier 2
EPHB1	EPH Receptor B1	3.68E-02	4.73E-07	1.32E-03	8.79E-02	78.00%	1.10E-03	tier 3
ALDH3A1	Aldehyde Dehydrogenase 3 Family Member A1	9.48E-02	7.84E-07	−4.16E-03	4.59E-01	82.60%	2.45E-04	tier 3
NUDT5	Nudix Hydrolase 5	1.46E-01	1.50E-06	3.87E-03	5.27E-02	92.30%	4.00E-01	tier 3
DCXR	Dicarbonyl/L-Xylulose Reductase	−1.09E-01	2.22E-06	−3.76E-03	5.07E-02	72.90%	4.58E-01	tier 3
IL10RB	Interleukin 10 Receptor Subunit Beta	−1.72E-01	2.49E-06	−7.64E-04	2.02E-01	97.40%	1.55E-02	tier 3
CCL11	C-C Motif Chemokine Ligand 11	1.11E-01	2.90E-06	4.31E-04	2.08E-04	99.30%	5.96E-02	tier 1
LYZ	Lysozyme	−1.84E-02	2.92E-06	−6.30E-04	4.05E-02	97.80%	1.91E-02	tier 1
SYT11	Synaptotagmin XI	−2.75E-01	4.28E-06	−8.93E-04	5.42E-01	62.50%	8.13E-01	tier 3
CCL25	C-C Motif Chemokine Ligand 25	2.55E-02	4.92E-06	6.91E-04	4.16E-02	85.80%	5.38E-01	tier 1
ITIH1	Inter-Alpha-Trypsin Inhibitor Heavy Chain 1	2.03E-02	5.58E-06	5.71E-04	6.38E-02	89.10%	5.40E-02	tier 3
ANXA2	Annexin A2	−3.56E-02	9.99E-06	−6.86E-04	1.66E-01	87.10%	1.48E-06	tier 3
IL9	Interleukin 9	−2.33E-01	1.54E-05	−1.05E-03	2.62E-01	91.40%	6.23E-04	tier 3

Among the 15 proteins, 10 demonstrated colocalization (PP.H4 > 0.6), while 12 (80%) showed strong evidence (PP.H4 > 0.8) (Supplementary Table S9). None were located within 500 kb of previously known CM GWAS loci. Additionally, eight proteins that passed MR and colocalization thresholds also showed associations with at least one CM risk factor (Supplementary Fig. S3), with 91% of these associations exhibiting directional concordance with CM risk factor findings.

### Genetic correlation estimates

Genetic correlation analysis revealed significant positive correlations between CM and ease of skin tanning and childhood sunburn occasions, supporting known CM risk factors. A suggestive negative correlation with caffeine intake was observed but did not reach statistical significance (Supplementary Tables S10, S11).

### Validation of identified proteins as prognostic biomarkers in CM

**Figure 4:**
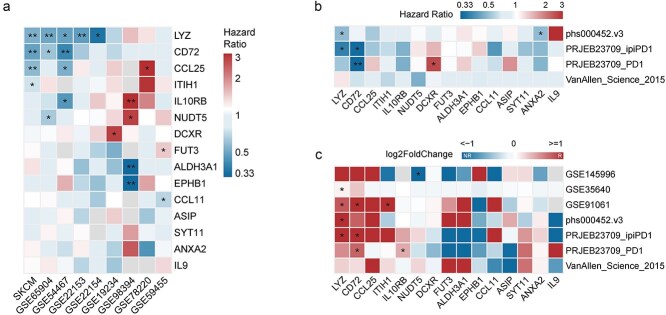
Validation in predictive and prognostic level of 15 proteins encoded genes in independent CM naïve and ICB cohorts. (a) Heatmap shows uni-cox regression hazard ratio (OS) and significance of 15 protein encoded genes in nine independent melanoma cohorts. (b) Heatmap shows uni-cox regression hazard ratio (PFS) and significance of 15 protein encoded genes in four independent melanoma ICB cohorts. (c) Heatmap shows log2FoldChange (NR versus R) and significance (Wilcoxon test) of 15 protein encoded genes in seven independent melanoma ICB cohorts with response status. ^*^P < .05, ^**^P < .01, ^***^P < .001, ^****^P < .0001.

The prognostic value of the 15 SMR-identified proteins was assessed across nine melanoma transcriptome datasets (Supplementary Table S1). Univariate Cox regression analysis revealed that LYZ, CD72, and CCL25 were consistently associated with overall survival, indicating their potential as CM prognostic markers (Fig. 4a). Additionally, ITIH1, IL10RB, NUDT5, DCXR, FUT3, ALDH3A1, EPHB1, and CCL11 were linked to survival outcomes in at least one dataset, warranting further validation.

To further investigate the shifting expression patterns of the identified genes during melanoma progression, we analyzed an additional dataset comprising samples from normal skin, benign nevus, primary melanoma, and metastatic melanoma (MM) (Supplementary Fig. S5, Table S12). Notably, several genes, including ANXA2, ALDH3A1, and EPHB1, exhibited a progressive increase in expression from normal skin and nevus to primary and MM, which is consistent with previous mendelian randomization and SMR results. In contrast, LYZ displayed elevated expression in nevus and primary melanoma but decreased levels in MM, aligning with its protective role identified through MR, SMR analysis, and Cox regression. Collectively, these findings reinforce the potential of the identified genes as both prognostic biomarkers and contributors to melanoma progression.

Given the role of ICB therapy in MM, we evaluated the predictive capacity of these proteins using seven independent ICB-treated melanoma cohorts with progression-free survival and treatment response profiles. LYZ and CD72 emerged as the strongest predictive biomarkers for ICB response, with high expression levels correlating with prolonged survival and improved treatment outcomes (Fig. 4b, c).

### Single-cell expression profiles of causal proteins in CM tissue

**Figure 5:**
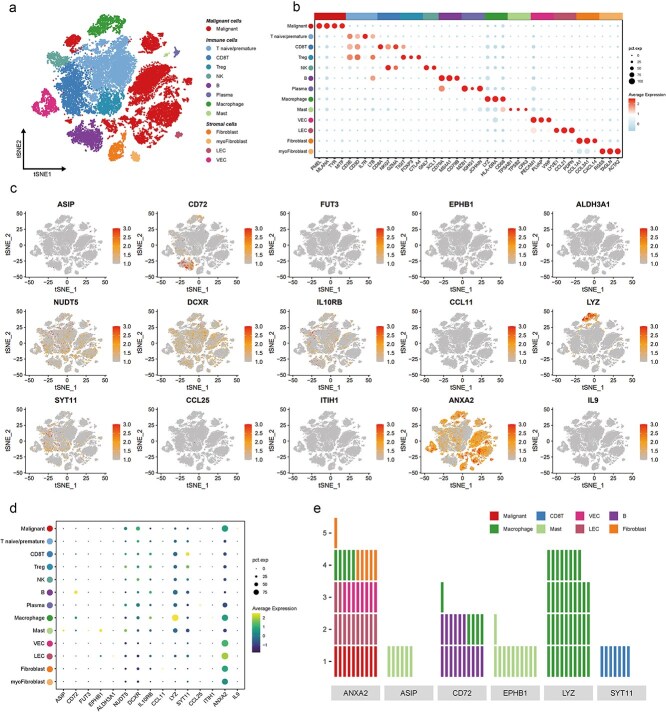
Celltypes specific expression of 15 proteins encoded genes in CM tumor tissue. (a) TSNE unsupervised dimensionality reduction of 60 CM tumor tissues from three datasets, showing 13 clusters with each cluster in different color. R package harmony was used to correct batch effects. (b) Dot plots showing average expression of known markers in indicated cell clusters. The dot size represents percent of cells expressing the genes in each cluster. (c) Expression levels of 15 protein encoded genes illustrated in TSNE plots. (d) Dot plots showing average expression of 15 protein encoded genes. The dot size represents percent of cells expressing the genes in each cluster. (e) Cell types specific expression of six proteins encoded genes with criteria of Log2FC > 0.5, FDR < 0.05 & pct. > 0.1 comparing with other celltypes. Each grid represents 0.1 average Log2FC.

To determine cell-type-specific expression patterns of these 15 proteins, we integrated three large-scale single-cell RNA-seq datasets (GEO), comprising 60 samples and 29 602 cells clustered into 13 distinct cell types (Fig. 5a). Cell-type markers confirmed clustering accuracy (Fig. 5b). Differential expression analysis identified cell-type specificity for several proteins (Log2FC > 0.5, FDR < 0.05, pct. > 10%) (Fig. 5e). Specifically, LYZ was enriched in macrophages, CD72 in B cells and macrophages, SYT11 in CD8+ T cells, EPHB1 and ASIP in mast cells, and ANXA2 in malignant cells (Fig. 5c, d, Supplementary Table S13).

### P‌PI network, pathway enrichment, and druggability analysis

PPI analysis revealed significant interactions, including LYZ-ANXA2 and IL10RB-IL9-CCL25-CCL11 (Supplementary Fig. S4a). Pathway enrichment analysis linked these proteins to keratinocyte function, immune response, and metabolic reprogramming (Supplementary Fig. S4b).

Druggability assessment classified EPHB1, IL10RB, CCL11, and IL9 as druggable targets, while CD72, LYZ, FUT3, ALDH3A1, NUDT5, DCXR, SYT11, CCL25, and ANXA2 were identified as targets of existing licensed or clinical-phase drugs. Using MR findings, we inferred potential pharmacological actions, noting that EPHB1, IL10RB, and CCL11 matched existing drug mechanisms, while IL9 may require an inhibitor rather than an agonist (Supplementary Table S14).

Molecular docking analysis of LYZ was performed to assess drug-binding affinity. Glyburide and cyclopenthiazide exhibited the strongest binding energies (−8.0 kcal/mol), suggesting their potential for CM treatment (Supplementary Figure 6SA-N, Supplementary Table S15). To investigate potential pharmacological strategies for activating CD72, we conducted structure-based virtual screening using 1609 FDA-approved compounds. Among them, Dihydroergotamine emerged as the top-ranked candidate, exhibiting the strongest predicted binding affinity to CD72 (−10.16 kcal/mol) (Supplementary Fig. S7, Supplementary Table S16), which highlight the potential for drug repurposing strategies targeting CD72 in melanoma treatment. To further assess the stability of the CD72–dihydroergotamine complex, we performed a 20 ns molecular dynamics simulation. The results showed that the complex rapidly reached equilibrium and maintained overall stability during the simulation (Supplementary Fig. S8a). Residue-level fluctuations were mainly confined to surface-exposed loop regions, while the binding pocket itself remained rigid (Supplementary Fig. S8b). Solvent accessibility at the protein–ligand interface was consistent across the trajectory, further supporting the persistence of the binding interaction (Supplementary Fig. S8c). The potential energy profile of the system remained stable without major perturbations, indicating a well-equilibrated complex (Supplementary Fig. S8d). Finally, stereochemical evaluation confirmed that the protein backbone conformations were largely restricted to favorable regions (Supplementary Fig. S8e). Collectively, these results demonstrate that dihydroergotamine forms a stable and energetically favorable interaction with CD72 under simulated physiological conditions.

### Mouse knockout models and safety assessments

MGI database queries identified knockout (KO) mouse models for 22 candidate genes (15 SMR proteins and 7 GWAS loci), with several (ALDH3A1, EPB41L1, LYZ, MTAP, ASIP) showing skin pigmentation abnormalities, delayed barrier formation, and altered coat color, suggesting intrinsic roles in melanogenesis and CM susceptibility (Supplementary Table S17).

Two-sample MR analyses assessed genetic associations between GWAS variants, SMR-identified proteins, and safety outcomes relevant to CM prevention trials. No significant associations (Bonferroni-corrected *P* < .003) were found with major safety traits including lung, prostate, colorectal, and breast cancer, chronic kidney disease, Alzheimer’s disease, coronary artery disease, or type 2 diabetes, apart from rs543069395 and rs56280801 (Supplementary Table S18).

## Discussion

Our genetic study, encompassing 651 324 individuals, identified seven novel genetic variants for cutaneous melanoma (CM) through GWAS and 15 putatively causal proteins via SMR and colocalization analyses using plasma pQTL data. These findings expand current knowledge on CM-related biological pathways and highlight CD72 and LYZ as promising drug targets for CM prevention and treatment.

Functional annotation of the seven novel genome-wide significant variants revealed plausible links to melanoma biology. Several loci mapped to genes involved in cell–cell adhesion (e.g. CADM2) [47], extracellular matrix interactions (e.g. COL13A1) [48], vesicular trafficking (e.g. EXOC6) [49], and membrane lipid asymmetry (e.g. ATP8B4) [50], all of which are processes relevant to melanoma cell invasion, immune evasion, and tumor–stroma communication. Others were located within or near non-coding RNAs (e.g. RP11-310H4.3, RP11-766 N7.3, SNORD65) [51, 52] that may act as *cis-*regulatory elements influencing transcriptional programs in melanocytes or immune cells. These annotations provide a mechanistic framework to interpret the statistical associations and may inform future functional studies.

In support of our findings, mouse model with deletion of ASIP and ALDH3A1 genes developed skin pigmentary abnormalities, such as increased skin pigmentation, delayed skin barrier formation, abnormal skin appearance, and darkened coat color. ASIP (Agouti Signaling Protein) plays a pivotal role in melanogenesis regulation. Mutations within the ASIP gene have been implicated in altering the severity of melanoma [53]. The binding of ASIP to the Melanocortin 1 Receptor (MC1R) inhibits alpha-MSH mediated signaling, thereby attenuating eumelanogenesis (production of brown/black pigment) and consequently augmenting the synthesis of pheomelanin (yellow/red pigment) [54, 55]. Notably, ASIP mutations have been linked with the development of facial pigmented lesions and associated with a heightened melanoma risk, as indicated by a 5-fold increase in the hazard of death from melanoma in the presence of the ASIP TG/TG diplotype [53]. ALDH3A1 (Aldehyde Dehydrogenase 3 Family Member A1) contributes significantly to the stemness and immunogenic profile of melanoma and NSCLC (Non-Small Cell Lung Cancer) cells. Overexpression of ALDH3A1 was found to enhance the secretion of PD-L1 in melanoma cells in vitro [56]. Furthermore, there is a consistent correlation between the levels of ALDH3A1 expression and those of PD-L1 and COX-2 in clinical samples [57], highlighting the significance of ALDH3A1 in the pathophysiology of melanoma. Through additional comparison between normal skin, benign nevus, primary melanoma, and MM, we found that previously protective putative gene LYZ expression in normal skin is relatively low, while both nevus and primary melanoma samples display elevated levels, which then markedly decrease in MM. This pattern suggests that LYZ may play a protective role during the early stages of melanocytic transformation and tumor progression, but its downregulation is associated with melanoma metastasis. This observation suggests that LYZ could serve as a novel dynamic network biomarker to monitor melanoma progression or predict metastasis.

Additional druggable genes identified were EPHB1, IL10RB, CCL11, and IL9. IL9 has been demonstrated to enhance the survival and function of human melanoma cells, increasing their cytotoxic activity against these cells [58, 59], which were confirmed in our analysis. Research [60] has shown that mice deficient in T helper type 17 (T(H)17) pathway genes, specifically those encoding retinoid-related orphan receptor γ and IL-23 receptor, produced abundant IL-9, which led to significant growth inhibition of B16F10 melanoma in these mice. Conversely, the use of IL-9-blocking antibodies reversed this tumor growth inhibition, enhancing tumor growth in wild-type mice. Similarly, Il9r(−/−) mice displayed accelerated tumor growth. However, administering recombinant IL-9 (rIL-9) to tumor-bearing wild-type and Rag1(−/−) mice inhibited the growth of both melanoma and lung carcinoma. The adoptive transfer of tumor-antigen-specific T(H)9 cells into these mice suppressed melanoma growth, an effect nullified by neutralizing antibodies to IL-9. Notably, exogenous rIL-9 inhibited tumor growth in Rag1(−/−) mice but not in mast-cell-deficient mice, indicating that IL-9 targets in this context include mast cells but not T or B cells. Further, a higher presence of T(H)9 cells was observed in normal human skin and blood compared to metastatic lesions in patients with progressive stage IV melanoma, suggesting a potential role for IL-9 in tumor immunity and highlighting avenues for therapeutic strategies.

In contrast to other proteins such as CCL11 and LYZ, whose roles in melanoma are relatively well-established, CD72 represents a less-characterized but promising target. CD72 functions as an inhibitory co-receptor on B cells and modulates immune responses. Our integrative genetic analyses revealed that higher CD72 levels are causally associated with reduced melanoma risk, suggesting a protective role, potentially through immunoregulatory pathways. Notably, our structure-based virtual screening identified Dihydroergotamine, an FDA-approved ergot alkaloid used for migraine management, as the top-ranked CD72-binding compound. While Dihydroergotamine has not been previously investigated in the context of melanoma, several studies have implicated ergot derivatives in modulating immune [61] and vascular responses [61], both of which are highly relevant to melanoma pathogenesis. In particular, Dihydroergotamine exhibits affinity for serotonin and adrenergic receptors, which are expressed in both the tumor microenvironment and immune effector cells [62]. Furthermore, recent studies suggest that Dihydroergotamine may affect cytokine signaling, tumor-associated macrophage polarization, and endothelial barrier function, indirectly impacting tumor immune evasion and metastatic potential [63–65]. These pleiotropic effects make Dihydroergotamine a biologically plausible candidate for repurposing in oncology, especially if it can modulate CD72 activity in immune cells within the tumor milieu. Taken together, our findings highlight CD72 as a novel immune-protective target in melanoma and identify Dihydroergotamine as a promising pharmacological agent for further evaluation. Future work will focus on molecular dynamics simulations, ligand binding validation, and functional assays to determine whether Dihydroergotamine can act as a CD72 agonist and suppress melanoma progression through immune modulation.

The strengths of the current analysis are multiple. First, the large number of CM cases included in our analysis led us to identify new variants and putatively causal genes for CM through GWAS and SMR proteomics. Second, we used three complementary strategies—nearest gene (local method), PoPs (global method), and pLoF to assign the most likely gene responsible for the GWAS signal with CM. Third, we provide biological credibility for most of our genetic findings through an extensive and complementary analysis covering CM risk factors. Fourth, in GTEx analysis, we showed that our proposed variants, in addition to associations with CM risk factors, were also associated with gene expression, and protein levels related to skin tissue. Fifth, KO models of 12 genes identified through GWAS and SMR developed highly relevant phenotypes to CM. Six, we systematically examined the associations between plasma protein biomarkers and CM risk by employing a two-stage proteome-wide MR design with the advantages of large sample sizes and minimal risk of reverse causation and confounding bias. The consistency of results among multiple rigorous analyses confirmed the robustness of the study findings. Additional evidence from single cell-type expression analysis, PPI, and druggability evaluation provided insights into the potential pathogenic effect of candidate proteins on CM and further prioritized druggable targets. Although the lack of drug information of several proteins (e.g. CD72 and LYZ), these proteins still deserve to be a promising new therapeutic target for CM. Seven, the lack of associations between the distinct GWAS loci and SMR genes with safety outcomes provides some reassurance on target safety profiles.

Nevertheless, several limitations of this study should also be considered. First, the current analysis was restricted to European populations. While this does reduce the potential bias caused by population stratification, our results may not apply to populations of other ancestral groups. Second, although it does not invalidate the results identified, the absence of CM subtypes in this analysis most certainly decreased our ability to detect signals specific to CM subtypes. Future genomic analysis should extend to different CM subtypes, as identify the genomic subtype of melanoma is an important requirement for the clinical management of melanoma patients. Third, we assessed the role of plasma proteins in CM but could not estimate the levels of relevant proteins in other tissues. Assessing the role of protein levels from other tissues in CM may provide more insight into CM pathogenesis, especially skin tissue. Fourth, the strict significance threshold and evidence grading criteria may lead to underestimation the convincing of the associated proteins. Furthermore, the current statistical analyses and strict significance threshold might filter out these plasma proteins that are ‘downstream’ of the ‘driver’ proteins. Further mechanistic studies are needed to uncover the ‘driver’ and ‘downstream’ proteins involved in CM onset and development. Finally, although we applied stringent variant filtering criteria (e.g. excluding variants with MAF < 0.005) and used a fixed-effects inverse-variance weighted meta-analysis to harmonize UK Biobank and FinnGen datasets, we acknowledge the potential for residual biases due to differences in imputation reference panels (HRC versus SISu v3). Such discrepancies may lead to subtle variation in allele frequency estimates or imputation quality between cohorts, which could affect the accuracy and comparability of the meta-analysis. While the consistency of our results across independent replication datasets provides some reassurance, future studies using harmonized imputation pipelines or raw genotype data may further reduce these uncertainties.

In conclusion, we discovered a total of seven distinct novel CM-associated variants and 15 causal proteins for CM through GWAS and SMR-proteomics with evidence of biological plausibility. The new mechanisms and pathways together with the protein druggability discovered provide a tractable path for the translation of our genomic findings for the development of screening biomarkers and therapeutic drugs for CM.

**Key Points**

- A large-scale genome-wide association studies meta-analysis of 651 324 individuals identified seven novel genetic loci associated with cutaneous melanoma risk.
- Proteome-wide Mendelian randomization and colocalization analyses uncovered 15 plasma proteins causally linked to melanoma susceptibility.
- Among these, CD72 and LYZ emerged as promising therapeutic targets, supported by prognostic and predictive value in multiple melanoma cohorts.
- Single-cell RNA sequencing revealed that these proteins are predominantly expressed in macrophages, B cells, CD8 T cells, and malignant cells, highlighting immune-tumor interactions.
- Drug repurposing analyses identified Dihydroergotamine as a candidate targeting CD72, and Glyburide/Cyclopenthiazide as potential LYZ modulators, providing a translational path for therapeutic development.

## Supplementary Material

Supplementary_Information_bbaf564

Supplementary_Table_bbaf564

## Data Availability

The summary data of GWAS meta-analysis can be downloaded from the website https://my.locuszoom.org/. The other datasets generated and/or analyzed during the current study are publicly available and included in this published article and its supplementary information files.
