# Supplementary material for: Genetic imputation of transcriptome and proteome illuminates novel therapeutic targets of cutaneous melanoma
Source: Brief Bioinform. 2025 Oct 28;26(5):bbaf564. doi: 10.1093/bib/bbaf564 (PMC12560784; doi:10.1093/bib/bbaf564)
Supplement: Supplementary_Information_bbaf564 [file supplementary_information_bbaf564.pdf]

Genetic imputation of transcriptome and proteome illuminates novel therapeutic targets of cutaneous melanoma

Supplementary methods

Genotyping, imputation, and quality control

Genotyping and imputation in the UKB have been described in detail elsewhere [1]. Briefly, two custom Affymetrix UKBileve and UKB Axiom arrays (designed to optimize imputation performance) were used for genotyping of DNA samples obtained from the UKB study participants. The UKB performed imputation centrally using an algorithm implemented in the IMPUTE2 program. Only markers that were present in both UKBileve and UKB Axiom arrays were used for imputation. To maximize the use of haplotypes with British and European ancestry, a special reference panel comprising a merged sample of UK10K sequencing and 1000 Genomes imputation reference panels was used for genotype imputation by the UKB. Genetic principal components to account for population stratification were computed centrally by UKB.

In FinnGen, genotyping of the samples was performed using Illumina and Affymetrix arrays (Illumina Inc., San Diego, and Thermo Fisher Scientific, Santa Clara, CA, USA). Sample quality control (QC) was performed to exclude individuals with high genotype missingness (>5%), ambiguous gender, excess heterozygosity (±4 SD) and non-Finnish ancestry. Regarding variant QC, all variants with low Hardy–Weinberg equilibrium (HWE) p value ($<1\times10^{-6}$), high missingness (>2%) and minor allele count (MAC) <3 were excluded. Chip genotyped samples were pre-phased with Eagle 2.3.5 with the number of conditioning haplotypes set to 20,000. Genotype imputation was carried out by using the Finnish population-specific SISu v3 reference panel with Beagle 4.1 (version 08Jun17.d8b) as described in the following protocol: dx.doi.org/10.17504/protocols.io.nmndc5e. In post-imputation QC, variants with imputation INFO <0.6 were excluded.

Further details for the study population, genotyping, and imputation information have been described elsewhere [2-5].

Polygenic Priority Score

In our study, we adopted the Polygenic Priority Score (PoPS) methodology for the purpose of gene prioritization. PoPS represents an innovative gene prioritization approach, which effectively identifies candidate causal genes by integrating GWAS summary statistics with diverse biological data sources, including gene expression profiles, biological pathways, and protein-protein interaction networks. The rationale behind selecting PoPS as the method of choice rests in its established capability to nominate causal genes at non-coding GWAS loci with a high degree of predictive confidence, surpassing the performance of alternative similarity-based or locus-based methodologies.

The PoPS analysis encompassed the following steps: Initially, we employed the MAGMA software to compute gene association statistics in the form of z-scores and gene-gene correlations, utilizing GWAS summary statistics and linkage disequilibrium (LD) information sourced from the 1000 Genomes Project. Subsequently, PoPS conducted marginal feature selection by leveraging MAGMA to perform enrichment analysis independently for each gene feature. The modeling framework employed generalized least squares (GLS), and exclusively those features attaining a nominal significance threshold ($P < 0.05$) were retained.

In the subsequent phase, PoPS executed a joint enrichment analysis, encompassing all selected features simultaneously within a "leave one chromosome out" (LOCO) framework. The complete list of gene features utilized by PoPS is provided in our repository accessible at https://github.com/FinucaneLab/gene_features. It is imperative to acknowledge that, despite the utilization of data from gene expression datasets, protein-protein interaction networks, and pathway databases, the PoPS model may not capture variants that operate through mechanisms beyond its scope.

Ultimately, PoPS calculated polygenic priority scores for each gene by fitting a joint

model for the enrichment of all selected features. It is worth noting that the PoPS score assigned to a gene remains independent of the GWAS data on the chromosome where the gene is physically situated. The PoPS analysis yielded scores for a total of 18,076 genes for GWAS datasets. We subsequently annotated our GWAS loci within a 500 kb genomic window using Ensembl genes and selected the gene exhibiting the highest PoPS score within the locus as the prioritized candidate.

Summary-based Mendelian randomization (SMR) analyses on plasma protein and CM

We obtained pQTLs from a genome-proteome-wide association study of plasma protein levels measured with 4,907 aptamers in 35,559 Icelanders [27]. Mendelian randomization requires meeting three core assumptions. As an extension of the MR concept, SMR was developed to estimate the pleiotropic association between genetically determined traits (e.g., gene expression, DNA methylation, or protein abundance as exposure) and complex traits of interest (e.g., disease phenotype as outcome) [28]. Here, we performed SMR using the Linux version 1.0.3 of SMR software in the command line using default options (https://yanglab.westlake.edu.cn/software/smr/#Overview).

Proteome-wide Mendelian randomization (MR) analysis

The protein quantitative trait loci (pQTLs) from the above-mentioned proteomic studies were used for the selection of genetic instruments. We mapped SNPs (single-nucleotide polymorphism) to human genome Build 37 (NCBI GRCh37) for unifying genomic coordinates. The following criteria were used to select instruments and proteins: (i) SNPs associated with any protein were selected ($P < 5\times10^{-8}$); (ii) the SNPs and proteins within the Major Histocompatibility Complex (MHC) region (chr6:25.5–34.0Mb) were excluded due to their complex linkage disequilibrium (LD) structure; (iii) the LD clumping was then conducted to identify independent pQTLs for each protein ($r^2 < 0.001$).

Both the “*Two Sample MR*” [29] and “*MR-PRESSO*” [30] packages in R software (version

4.2.1) were used to estimate the MR and sensitivity findings. Inverse variance weighting (IVW) [31] was the main method to obtaining a global estimate as long as sensitivity analysis was successful. Additionally, we employed the MR robust adjusted profile score (MR.RAPS) [32] and maximum likelihood estimator (MLE) [33] and to validate MR results. MR.RAPS was calculated for the causal estimate to provide higher statistical power if weak IVs (instrumental variables) existence. The MR findings were presented using odds ratio (OR) and confidence interval (CI). MLE assumes the linear correlation of proteins and CM with normal distribution and allows for uncertainty. Assuming a significance level of $P < 0.05$, we inferred the possibility of a causal relationship.

Three sensitivity analyses, including the weighted median [34], MR-Egger [35] and MR-PRESSO [30] approaches, were performed. Assuming that at least 50% of the SNPs are valid, the weighted median method can generate consistent causal estimates [34]. The MR-Egger regression can detect and correct for possible pleiotropy, and the *P* value of the intercept > 0.05 indicates no horizontal pleiotropic effects [35]. The MR-PRESSO method can detect outliers and provide a causal estimate after the removal of corresponding outliers [30]. The strength of IVs was assessed by calculating the F-statistic using the formula:

$$F = \frac{R^2 \times (N-1-K)}{(1-R^2) \times K},$$

where R^2 represents the proportion of variance in the exposure explained by the genetic variants, N represents sample size, and K represents the number of instrument. F-statistic >10 was considered that there was no significant weak instrumental bias. The F statistic was calculated to measure the strength of instrument in the analyses in our GWAS meta-analysis given a large sample overlap between the exposure and outcome data [36]. To determine heterogeneity, we employed Cochran's Q test [37]. After completing the analysis, the leave-one-out test was used to evaluate the results' robustness further.

Colocalization analyses

To refine the results, we performed another Bayesian test for the colocalization of two traits using the *coloc* R package [38] (https://chr1swallace.github.io/coloc/, version 5.1.0) to estimate the posterior probability of shared variants. For each leading SNP in our GWAS meta-analysis, all SNPs within 100 kb up and downstream of the leading SNPs were retrieved for colocalization analysis to analyze the posterior probability of H4 (PP.H4), and PP.H4 > 0.8 is the well-applied cut-off for the evidence of colocalization of the GWAS and QTL association.

Associations of CM GWAS variants and SMR identified proteins with CM risk factors and safety traits.

For novel genetic variants and SMR identified proteins, we determined genetic associations for 9 CM risk factors and 9 safety traits. GWAS summary data on CM risk factors and safety traits was obtained from European-descent GWAS studies: risk factors including educational attainment (years of education) [23], smoking [23], alcohol consumption [39], childhood sunburn occasions [19], job involves heavy manual or physical work [19], facial ageing [19], coffee intake [19], ease of skin tanning [19], and average total household income before tax [19]; safety traits including cancers (lung [40], prostate [40], colorectal [40], breast [40]), chronic kidney disease [41], Alzheimer's disease [42], CAD [43], T2DM [44], or creatinine [45] . We further evaluated whether the directionality of CM risk factors associations was concordant with findings on CM; for example, for a variant that showed an increased risk of CM, we expect a positive association with a deleterious risk factor.

Querying the MGI database

We queried the Mouse Genome Informatics (MGI, http://www.informatics.jax.org/) resource for all candidate genes from our novel GWAS hits list or those suggested as causal from our MR/colocalization approach. MGI uses a standardized nomenclature, and controlled vocabularies such as the Mouse Developmental Anatomy Ontology, the Mammalian Phenotype Ontology, and the Gene Ontologies. As MGI extracts and organizes data from the primary literature, we have parsed all system abnormalities

associated with models on all the queried genes.

Druggability annotations

Proteins encoded by genes identified in the GWAS and SMR analyses for CM were annotated with drug tractability information based on information provided by OpenTargets (release 2021-03-08) [46]. The OpenTargets framework classifies potential drug targets into nine distinct, non-overlapping categories, termed 'buckets', according to the drug type and the stage within the drug discovery pipeline. Licensed drugs (bucket-1) including antibodies, small molecules, and other modalities; Drugs in clinical development (buckets 2 and 3) also covering antibodies, small molecules, and other modalities; Compounds in the preclinical phase (buckets 4 and 5) specific to small molecules; and Predicted druggable targets (buckets 6 to 8 for small molecules and buckets 4 and 5 for antibodies). Proteins not fitting these criteria were deemed non-druggable.

Non-druggable proteins were annotated with drug tractability information based on information provided by Drug Signatures Database (DSigDB, http://dsigdb.tanlab.org/DSigDBv1.0/) [47]. Specifically, with 22,527 gene sets and 17,389 distinct compounds spanning 19,531 genes, DSigDB is a sizable database that connects medicines and other chemicals to their target genes.

For genes that were the target or potential target of licensed drugs, we checked whether the disease indication was also a risk factor for CM, as this may introduce a bias analogous to confounding by indication in MR.

Survival and immunotherapy response validation in melanoma transcriptomic cohort.

The TCGA-SKCM gene expression data and clinical information were downloaded from The Cancer Genome Atlas, Genomic Data Commons Data Portal (GDC DataPortal), RRID:SCR_014514) (https://portal.gdc.cancer.gov/). Multiple independent cutaneous melanoma cohorts were acquired, consist of 8 treatment-naive

and 7 immunotherapy cohorts (Table S2). These data were collected from the Gene Expression Omnibus (GEO) (https://www.ncbi.nlm.nih.gov/geo/), the Sequence Read Archive (SRA) (https://www.ncbi.nlm.nih.gov/bioproject/) and the database of Genotypes and Phenotypes (dbGaP) (https://www.ncbi.nlm.nih.gov/gap/).

Single-cell transcriptomic validation and celltype specific expression analysis

The cell type-specific expression of encoded genes with evidence for a potential causal effect on cutaneous melanoma at the plasma protein levels was further evaluated by employing single-cell RNA-seq data of integrated datasets. Single-cell RNA-seq profiles of cutaneous melanoma including GSE115978, GSE72056 and GSE189889 was acquired from the Gene Expression Omnibus. We used the R package *"Harmony"* for single-cell datasets integration. Finally, the integrated dataset included 34569 genes across 29602 cells. The R package *"Seurat"* (Version 4.0.4) was used to perform further single-cell transcriptomic analysis. To examine whether the encoded genes were highly expressed in a certain celltype, the differential expression analysis based on the Wilcoxon rank-sum test was performed to compare gene expression levels between a specific celltype and the rest of the others. The genes with an average Log2 fold change (Log2FC) more than 0.5, a false discovery rate (FDR) less than 0.05 and expression proportion (pct.) more than 10% were identified as enrichment genes.

Supplementary Results

Gene-based association and pathway enrichment analysis

Gene-based association using GWAS summary statistics form our GWAS meta-analysis based on positional, eQTL, or chromatin interaction additionally identified genes passing study-wide significance as being associated with CM (Figure 2B, Supplementary Table S6). According to previous report [49], Polygenic Priority Score (PoPs) and the closest gene individually outperform other gene prioritization methods, therefore, we determined the gene closest to the indexed SNP, as well as the gene with the highest score from PoPs within a 500KB region, which take genome-wide features into account and providing complementary information for annotation of indexed

variants (Supplementary TableS2). For all the fine-mapping genes and PoPS suggested genes, we retrieved the results from gene-burden tests using putative Loss-of-Function (pLoF) variants from the Genebass-UK Biobank resource.

Based on CM GWAS, we conducted gene pathway enrichment analysis. After restricting the analysis to pathways described in Gene Ontology, KEGG, and Reactome, we observed 3 significant enriched pathways (FDR <5%). Specifically, biological pathways include melanin biosynthesis (adjusted P value < 0.001), interferon signaling pathway (P = 0.001), autophagy of mitochondrion (P = 0.001), T helper 17 cell differentiation (P = 0.001), and response to interleukin 4 (P = 0.002) were identified, details on Supplementary Table S9.

Genetic correlation estimates

Estimates of the genetic correlation between CM and CM risk factors are reported in Supplementary TableS13. Results that pass multiple testing at 5% FDR are denoted, including a positive genetic correlation between CM and Ease of skin tanning and with Childhood sunburn occasions, as well as a negative genetic trend between caffeine intake and CM, although this effect was not significant. (Supplementary Table S13).

SMR analysis identified 15 casual protein biomarkers for CM

To elucidate the functional relationships between genome-wide cis-pQTLs and CM, we employed SMR to assess the associations using the GWAS data on Icelandic Cancer Project and deCODE genetics. We conducted several analyses to minimize confounding and biases. For the SMR results that passed our significance threshold [PSMR<1.187 × 10^{-5} (Bonferroni correction, P < 0.05/4212)] (Figure 3A and B), we performed genetic colocalization analysis to ensure the SMR results were unlikely to be confounded by linkage disequilibrium (LD). (Figure 3C and Supplementary Table S10). We further used the HEIDI approach to test against the null hypothesis that the association detected by the SMR test is due to pleiotropy (*P HEIDI* >0.01). To further verify the observed findings, we performed two-sample MR for 15 proteins with GWAS

summary statistics from external cohorts. The effect of all proteins is shown in Supplementary TableS11. Among them, 6 proteins passed the MR test. We also conducted co-localization analysis, specifically, 15 proteins which passed SMR threshold showed suggestive evidence of colocalization between CM and pQTL signals (posterior probably of Hypothesis 4 (*PP.H4*): one common causal variant >0.6) and of which 12 proteins (80%) show strong evidence of colocalization (*PP.H4* >0.8), see details in Supplementary Table S10. None of the proteins in SMR results that colocalized was within 500 KB of a known CM GWAS loci. Besides, we further conducted two-sample MR analyses against CM risk factors. The results showed that 7 of 15 proteins showed a significant association with at least one of the CM risk factors (Supplementary FigureS3 and Supplementary TableS12).

Supplementary Figure

Supplementary Figure S1. Quantile-quantile (Q-Q) plot of CM GWAS from SNP-level (A), and gene-level (B).

Supplementary Figure S2. Locus zoom plots for the 7 novel CM GWAS-associated loci.

Supplementary Figure S3. Heatmap shows genetic association with known CM risk factors of 15 biomarker proteins in SMR estimation.

Supplementary Figure S4: PPI analysis and pathway enrichment.

Supplementary Figure S5. Differential expression of causal protein-encoding genes across melanoma progression.

Supplementary Figure S6. Docking results of CD72 (Cluster of Differentiation 72)/LYZ (Lysozyme) and potential drugs.

Supplementary Figure S7.Molecular docking analysis of 1,609 FDA-approved drugs with CD72.

Supplementary Figure S1. Quantile-quantile (Q-Q) plot of CM GWAS from SNP-level (A), and gene-level (B). The Q-Q plot demonstrates the number and magnitude of observed associations between genotyped SNPs and CM, compared to the expected association statistics under the null hypothesis that there is no association. The identity line is shown in red. Observed association statistics (y-axis) and expected association statistics (x-axis) are on a $-log_{10}P$ scale.

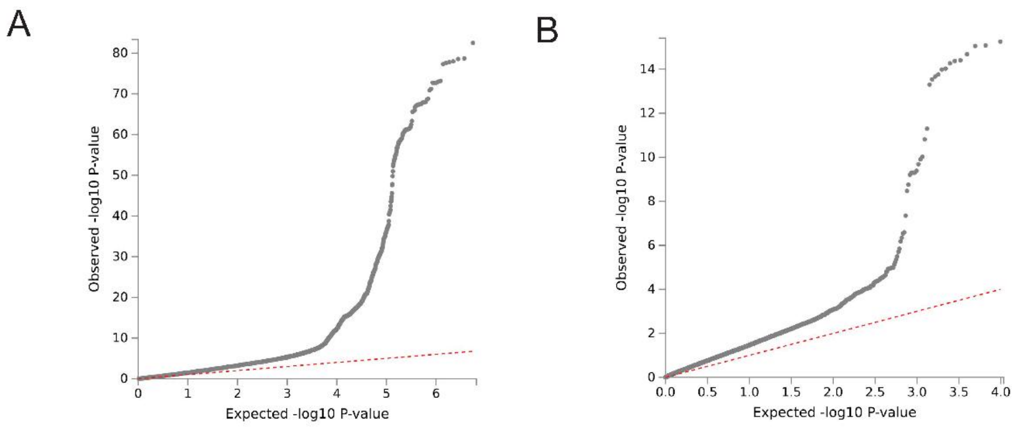

Supplementary Figure S2. Locus zoom plots for the 7 novel CM GWAS-associated loci. The plots illustrate fine mapping of the regions flanking each of the novel CM GWAS-associated loci. (rs41286740 were missing from hg19/1000 Genomes Nov 2014 EUR) The color coding indicates linkage disequilibrium of each SNP as measured by r^2 according to the shown scale with the relevant GWAS SNP annotated.

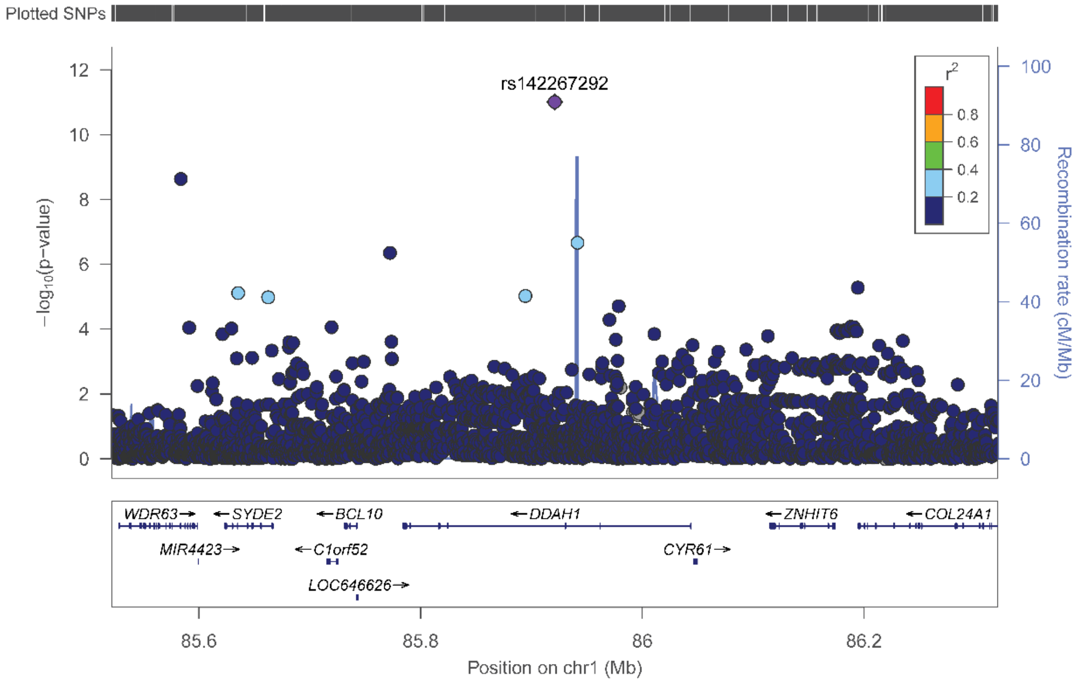

Supplementary Figure S2(A). Locus zoom plot for rs142267292.

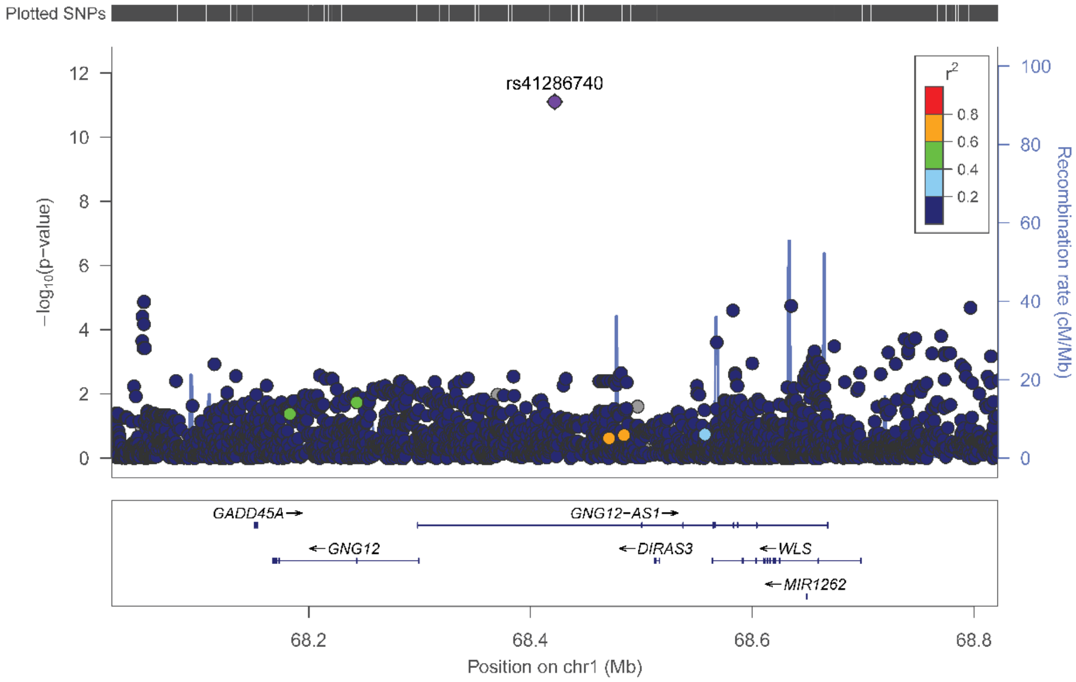

Supplementary Figure S2(B). Locus zoom plot for rs41286740.

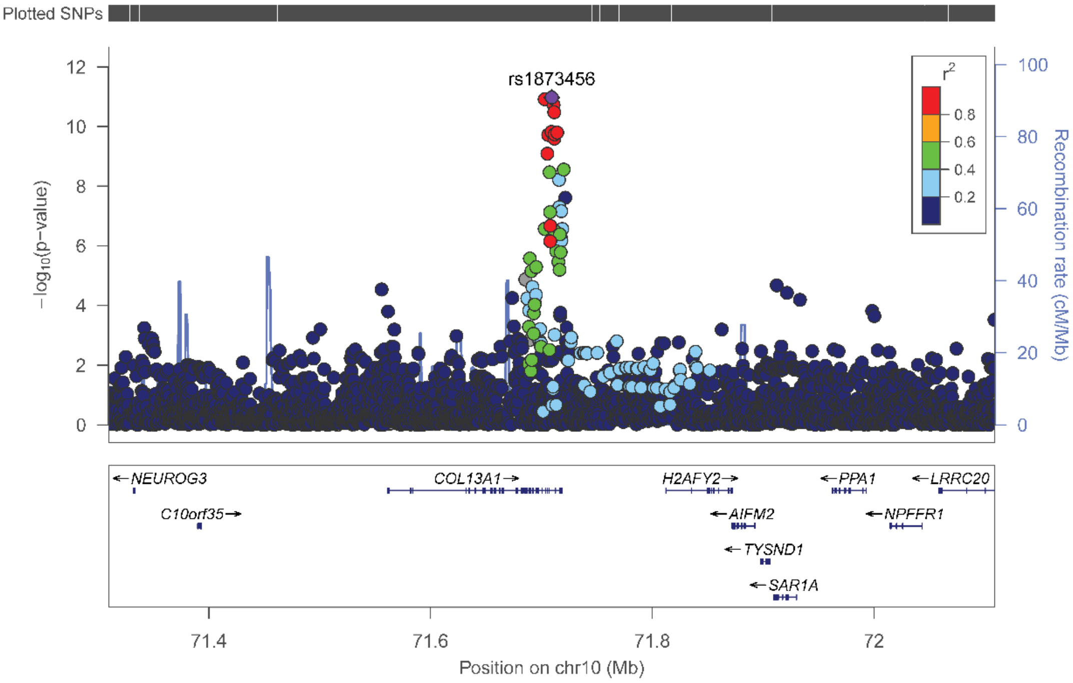

Supplementary Figure S2(C). Locus zoom plot for rs1873456.

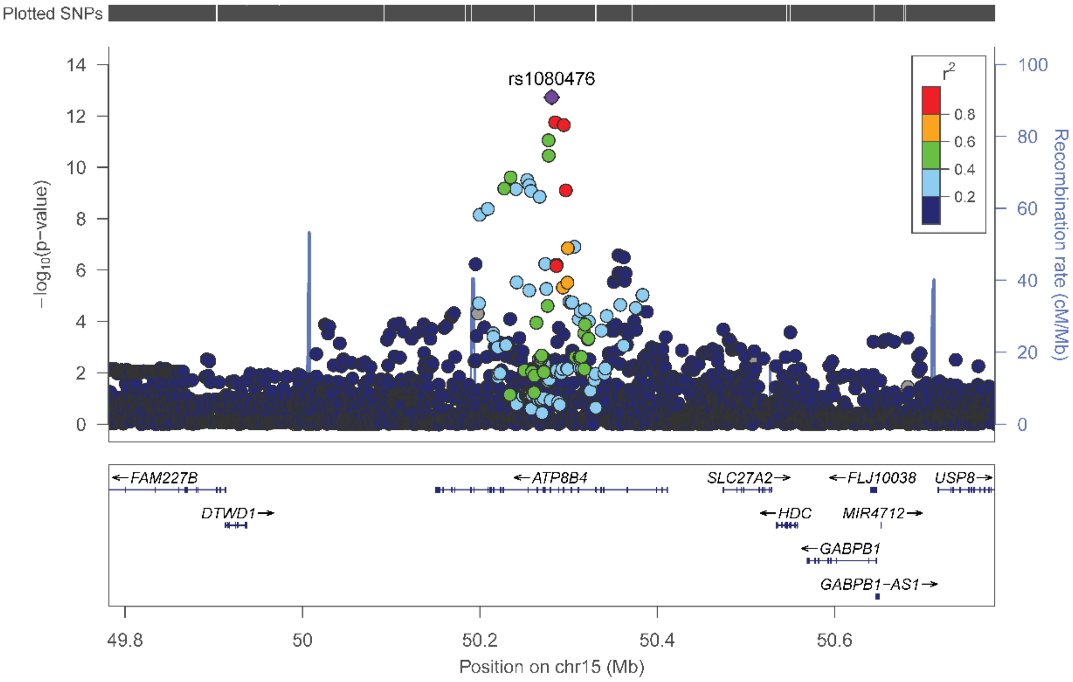

Supplementary Figure S2(D). Locus zoom plot for rs1080476.

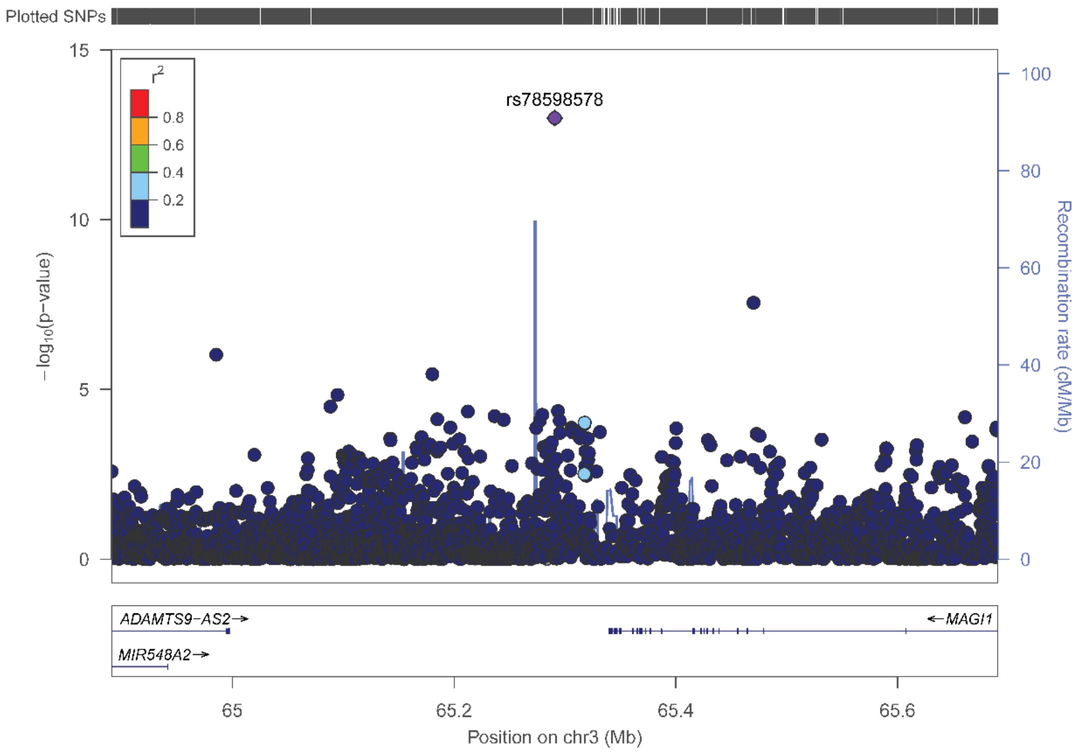

Supplementary Figure S2(E). Locus zoom plot for rs78598578.

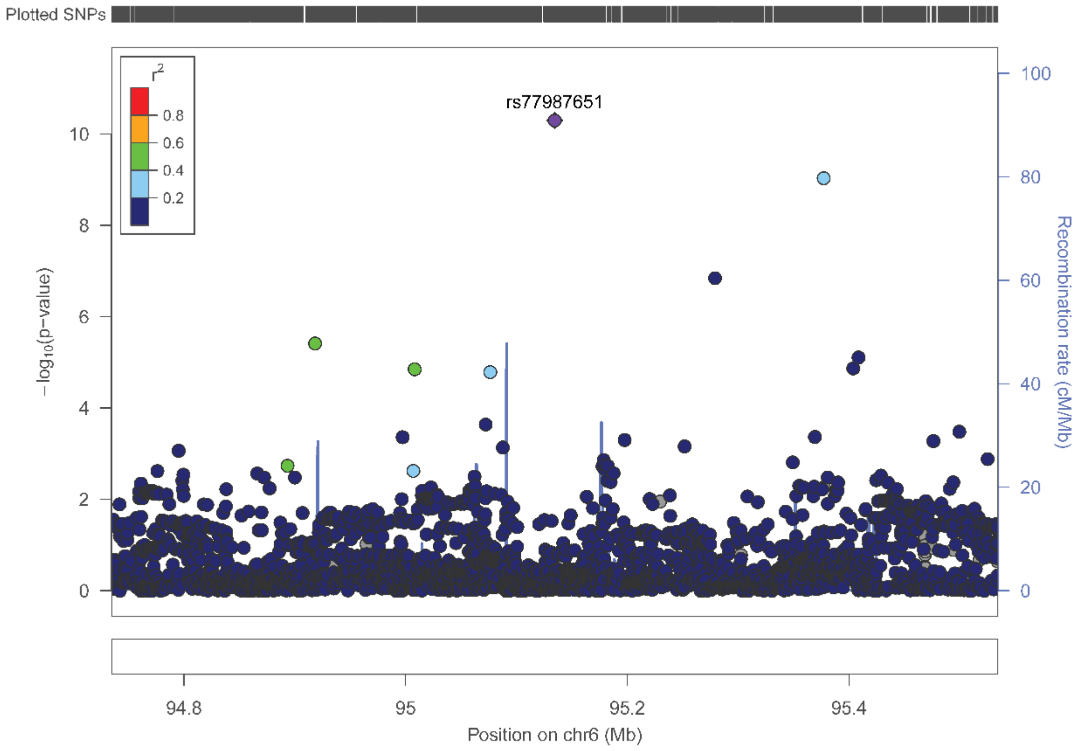

Supplementary Figure S2(F). Locus zoom plot for rs77987651.

Supplementary Figure S3. Heatmap shows genetic association with known CM risk factors of 15 biomarker proteins in SMR estimation. The color corresponds to the beta coefficient derived from MR. Blue corresponds to a negative association and red corresponds to a positive association; note that a positive β indicates either an increase in protein levels corresponding to an increase in CM risk or a decrease in protein levels corresponding to a decrease in CM risk, while a negative β indicates either a decrease in protein levels corresponding to an increase in CM risk or an increase in protein levels corresponding to a decrease in CM risk. *P < 0.05, **P < 0.01, ***P < 0.001, ****P < 0.0001

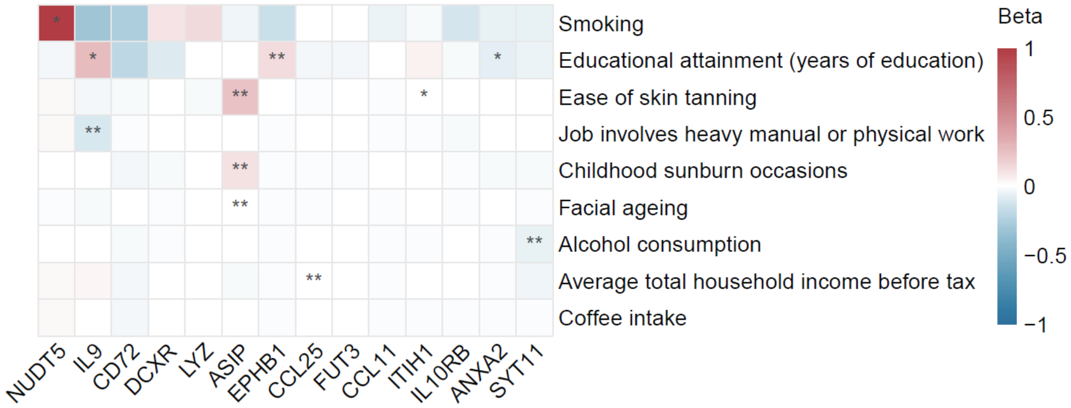

Supplementary Figure S4: PPI analysis and pathway enrichment.

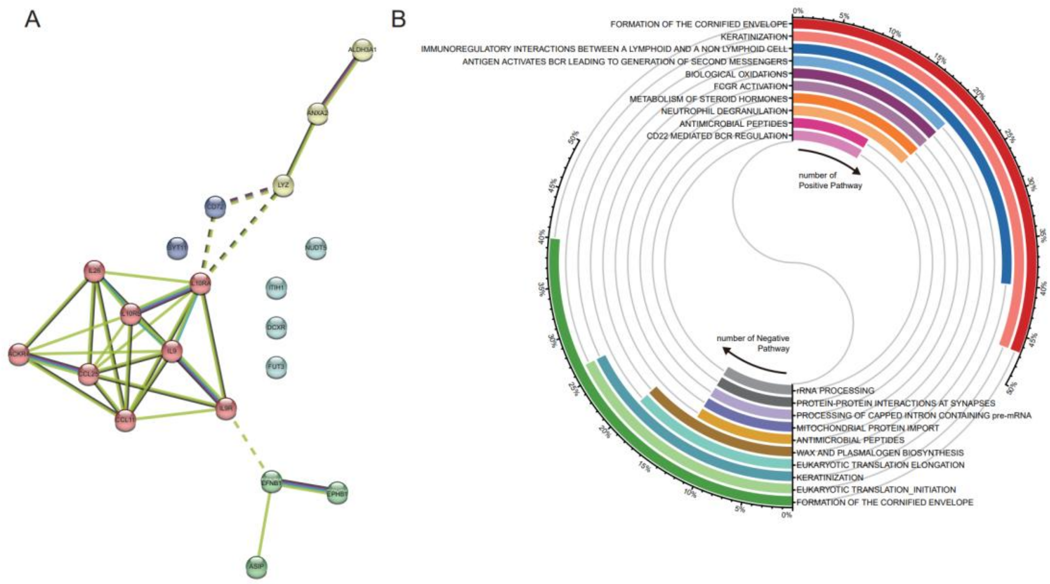

Supplementary Figure S4 (A) PPI analysis using String database.

Supplementary Figure S4 (B) Reactome pathway enrichment.

Supplementary Figure S5. Differential expression of causal protein-encoding genes across melanoma progression.

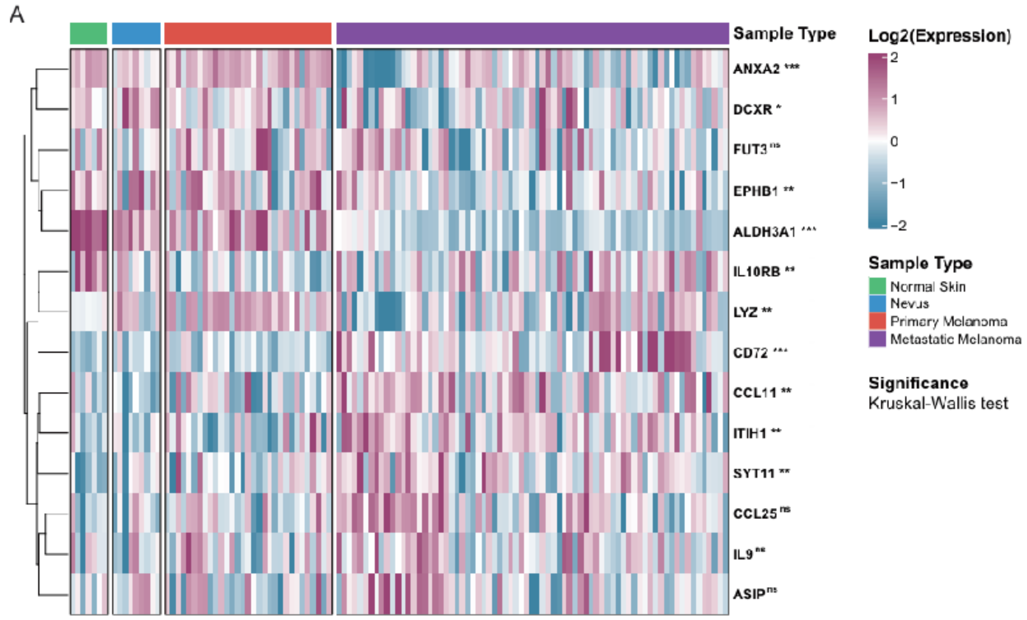

Supplementary Figure S5(A) Heatmap showing the expression levels of causal protein-encoding genes in samples from normal skin (green), nevus (blue), primary melanoma (red), and metastatic melanoma (purple).

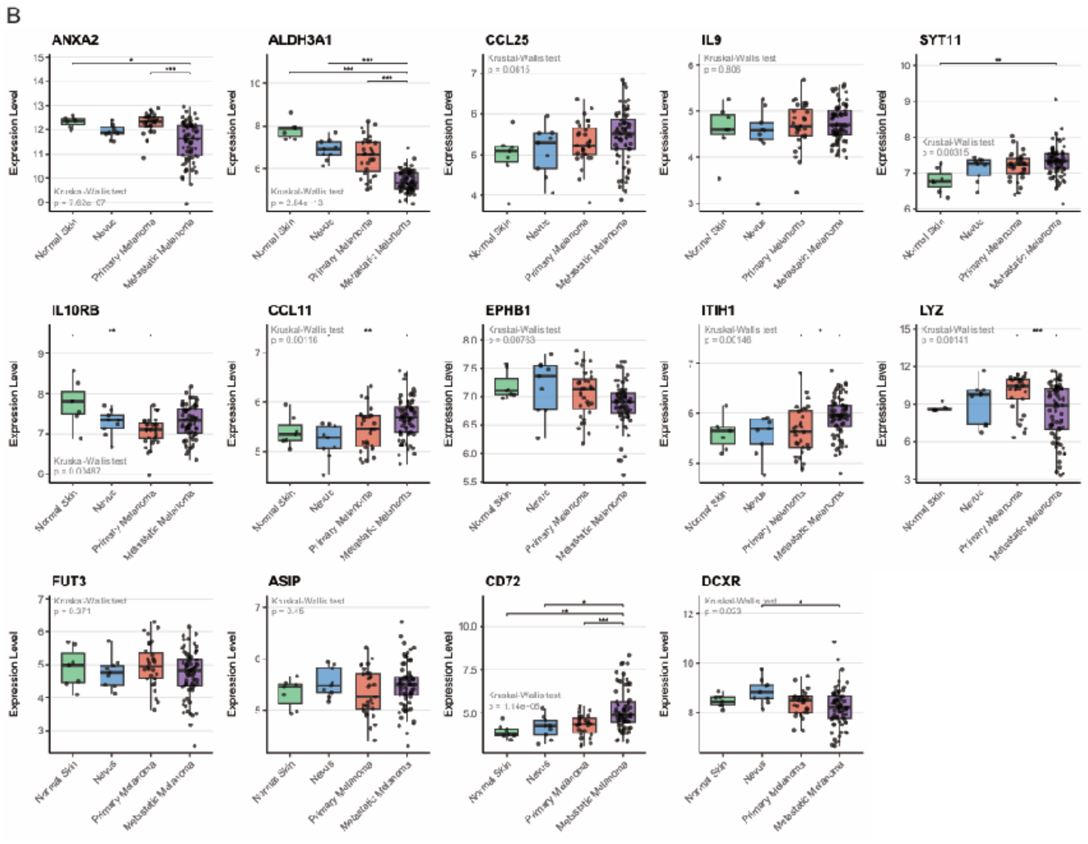

Supplementary Figure S5(B) Boxplots illustrating the expression levels of each gene across the four sample types. Statistical significance was assessed using the Kruskal-Wallis test (*p < 0.05, **p < 0.01, ***p < 0.001).

Supplementary Figure S6. Docking results of CD72 (Cluster of Differentiation 72)/LYZ (Lysozyme) and potential drugs.

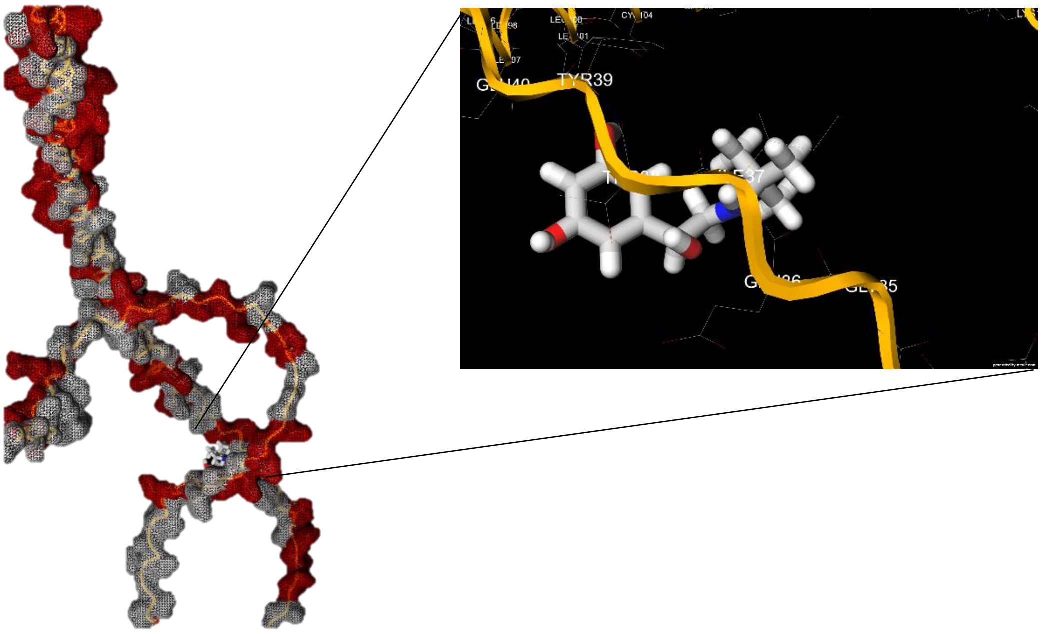

Supplementary Figure S6(A). Docking results of CD72 (Cluster of Differentiation 72) and Terbutaline.

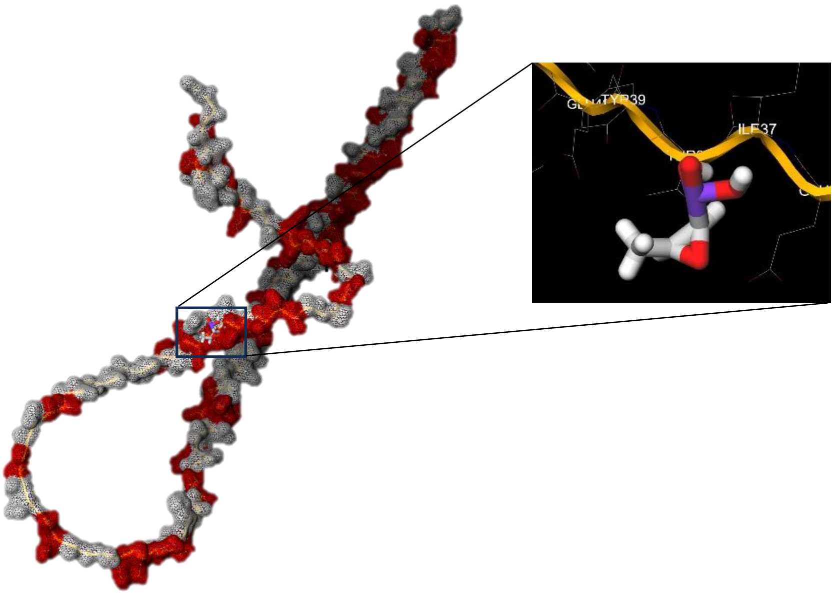

Supplementary Figure S6(B). Docking results of CD72 (Cluster of Differentiation 72) and Fosfomycin.

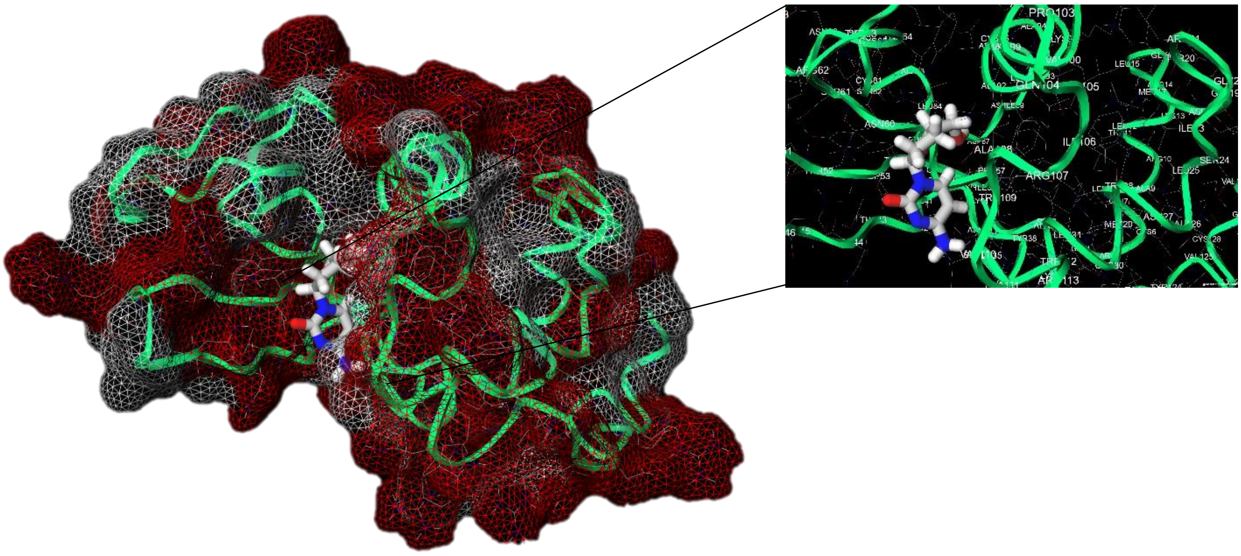

Supplementary Figure S6(C). Docking results of LYZ (Lysozyme) and Zalcitabine.

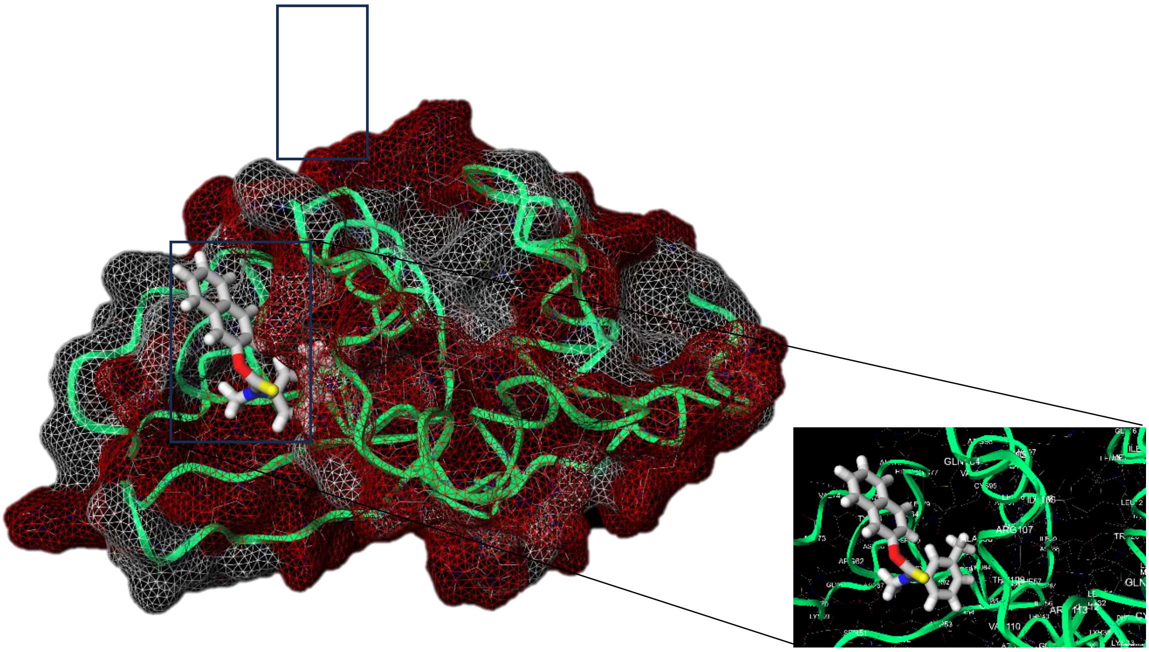

Supplementary Figure S6(D). Docking results of LYZ (Lysozyme) and Tolnaftate.

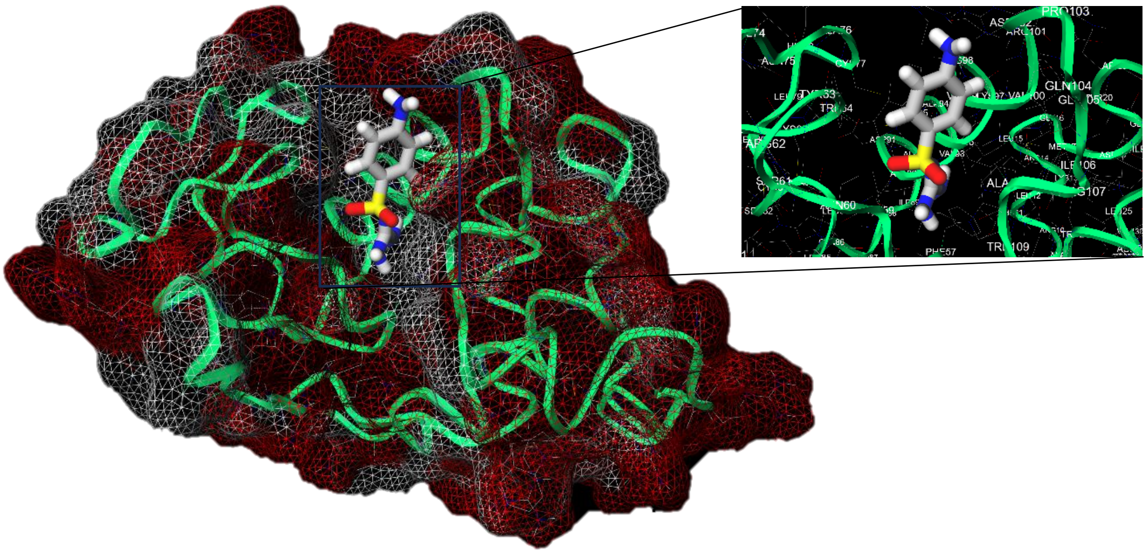

Supplementary Figure S6(E). Docking results of LYZ (Lysozyme) and Sulfaguanidine.

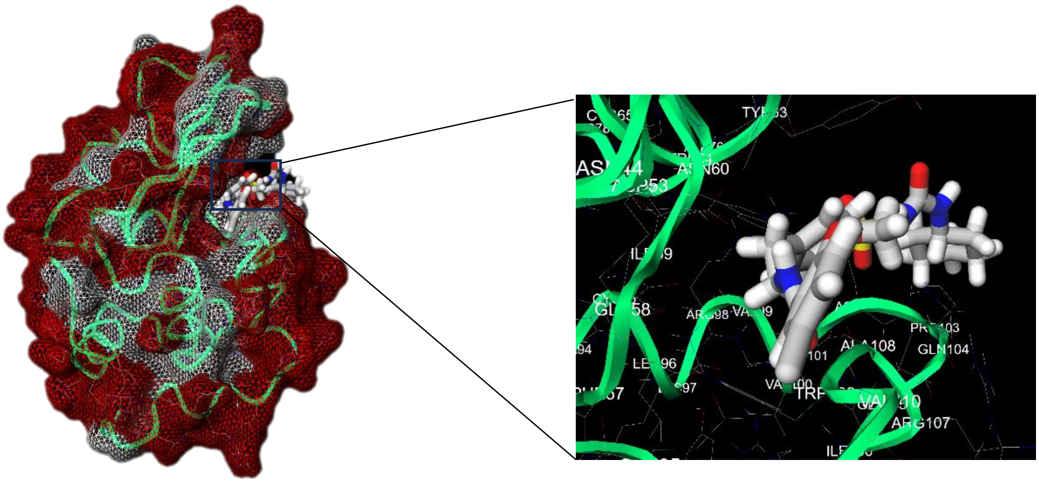

Supplementary Figure S6(F). Docking results of LYZ (Lysozyme) and Glyburide.

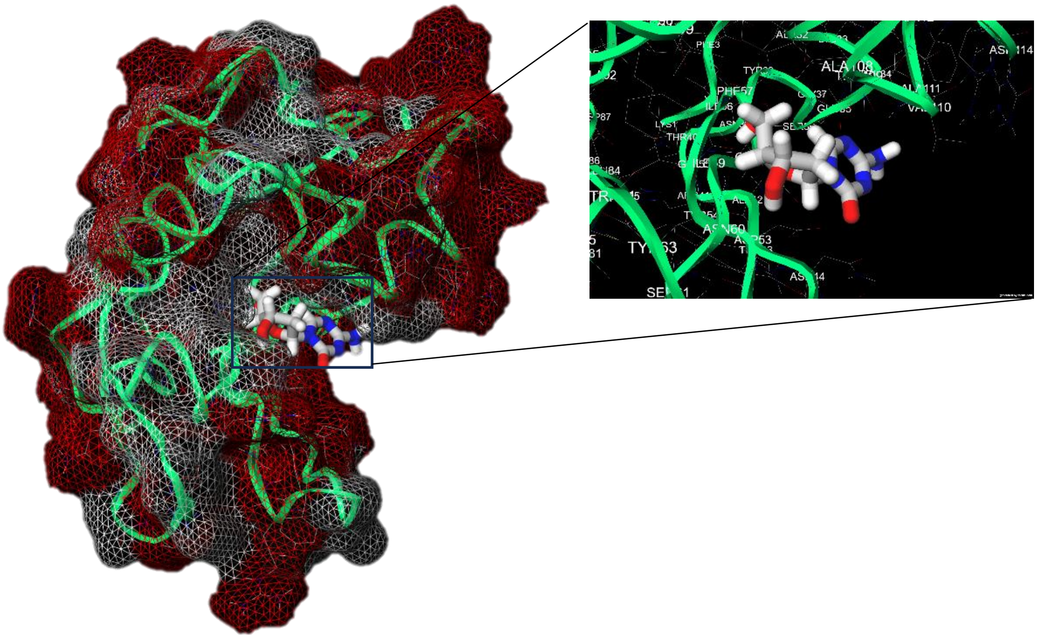

Supplementary Figure S6(G). Docking results of LYZ (Lysozyme) and Decitabine.

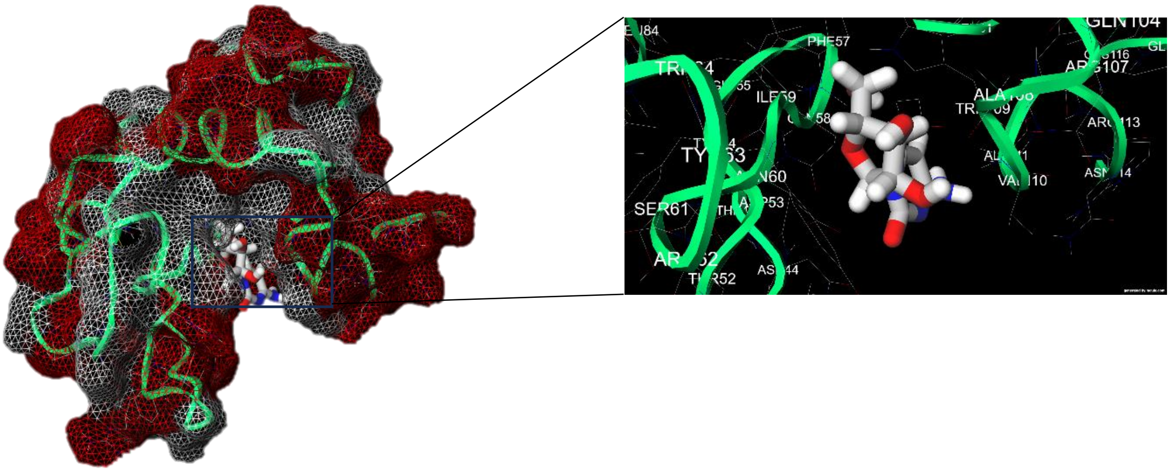

Supplementary Figure S6(H). Docking results of LYZ (Lysozyme) and Cytarabine.

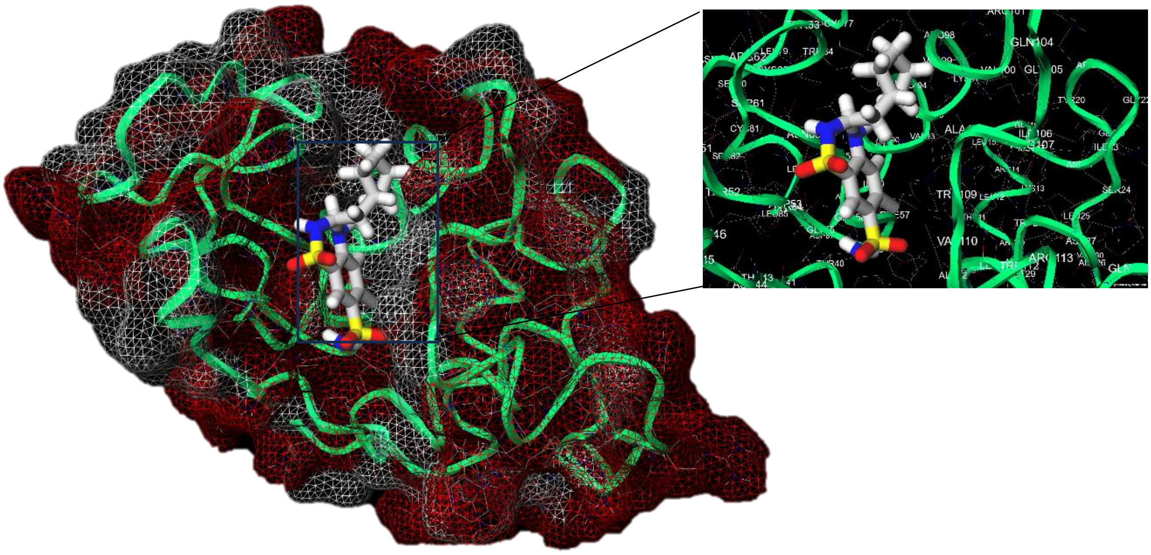

Supplementary Figure S6(I). Docking results of LYZ (Lysozyme) and cyclopenthiazide.

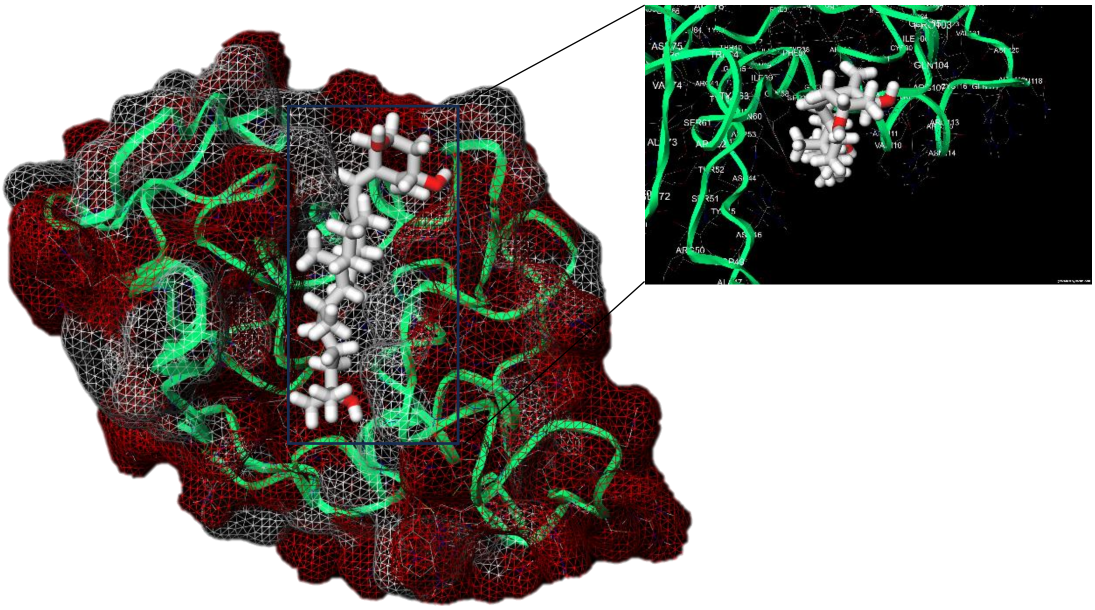

Supplementary Figure S6(J). Docking results of LYZ (Lysozyme) and Calcitriol.

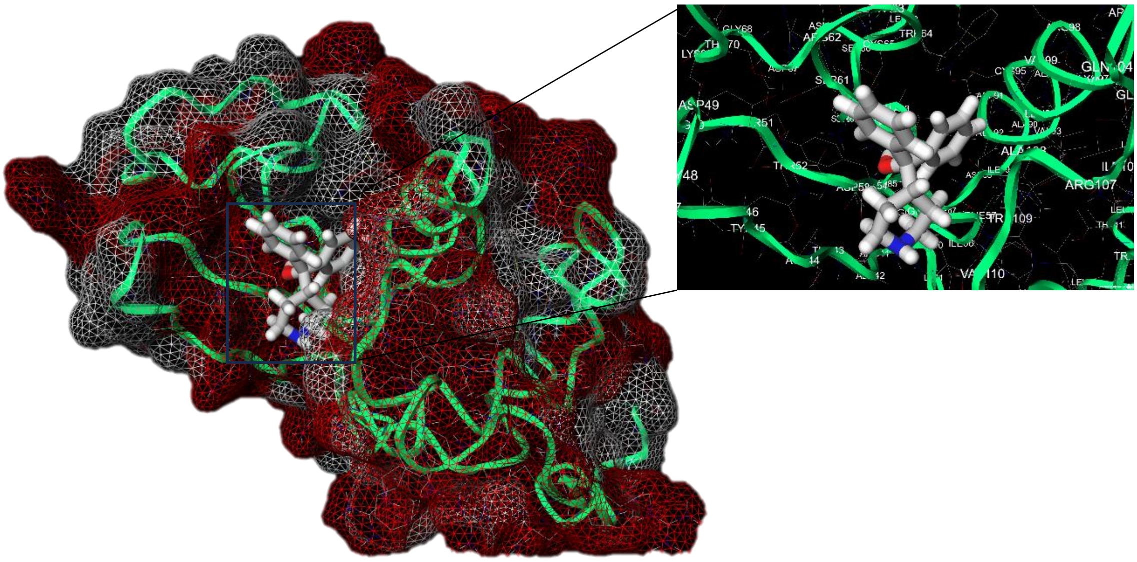

Supplementary Figure S6(K). Docking results of LYZ (Lysozyme) and Azacyclonol.

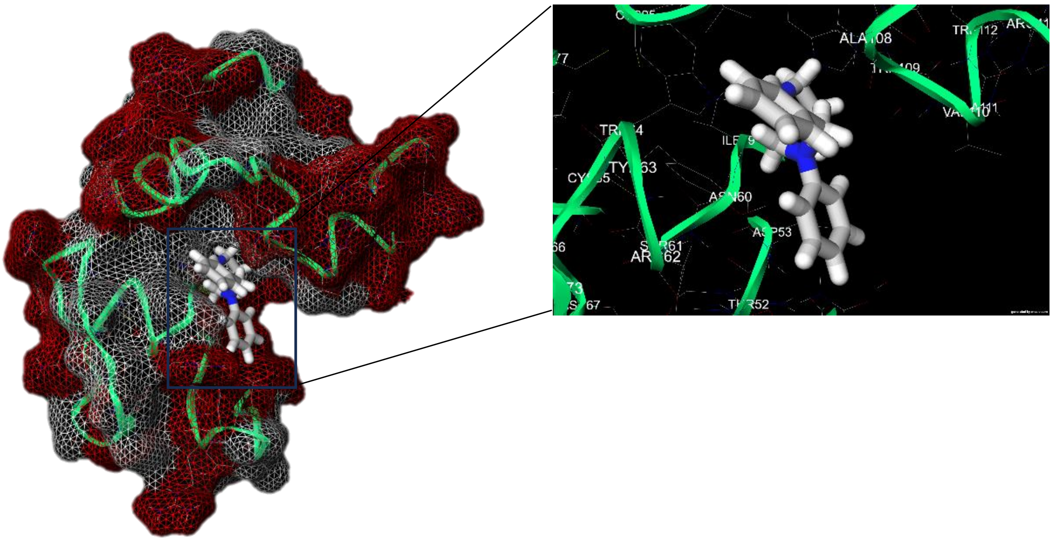

Supplementary Figure S6(L). Docking results of LYZ (Lysozyme) and Antazoline.

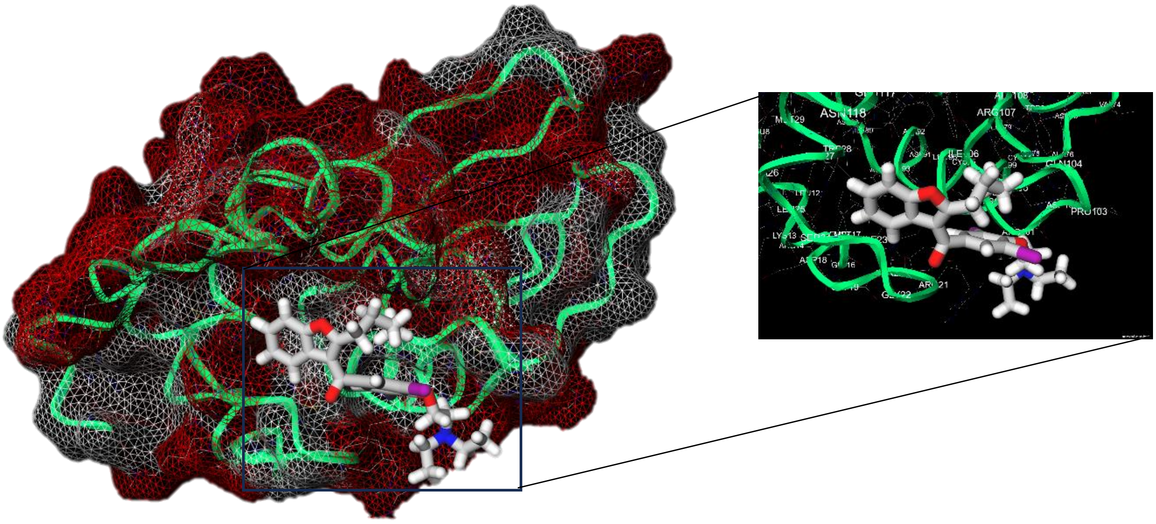

Supplementary Figure S6(M). Docking results of LYZ (Lysozyme) and Amiodarone.

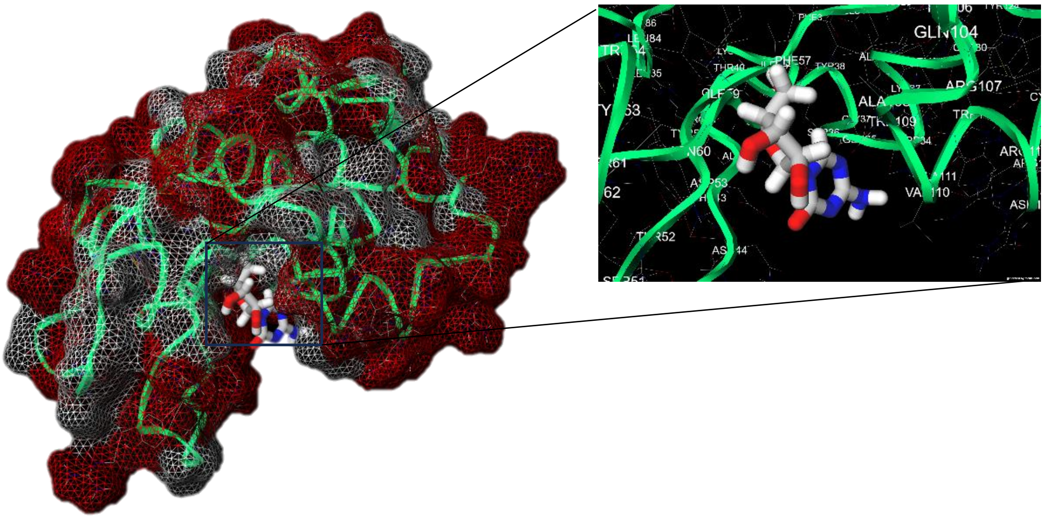

Supplementary Figure S6(N). Docking results of LYZ (Lysozyme) and 5-Azacytidine.

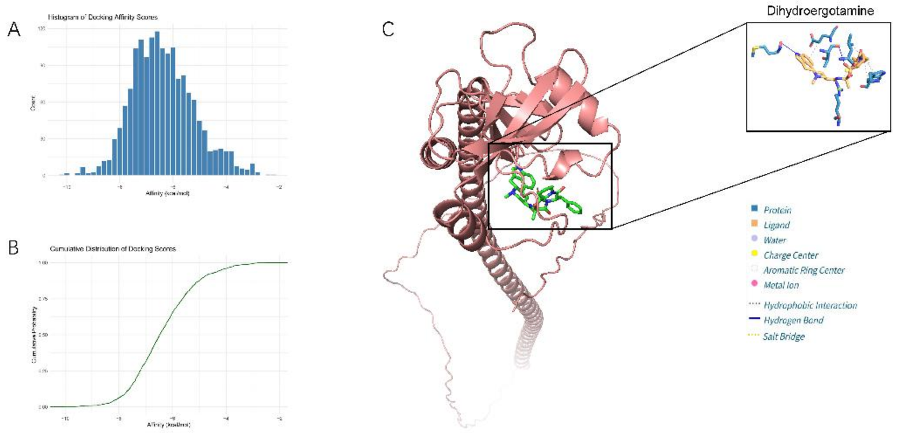

Supplementary Figure S7. Molecular docking analysis of 1,609 FDA-approved drugs with CD72.

(A) Histogram showing the distribution of docking affinity scores (kcal/mol) for all screened compounds.

(B) Cumulative probability plot of docking affinity scores, illustrating the proportion of compounds achieving a given binding energy threshold.

(C) Predicted binding mode of the top-ranked compound, dihydroergotamine, within the CD72 binding pocket.

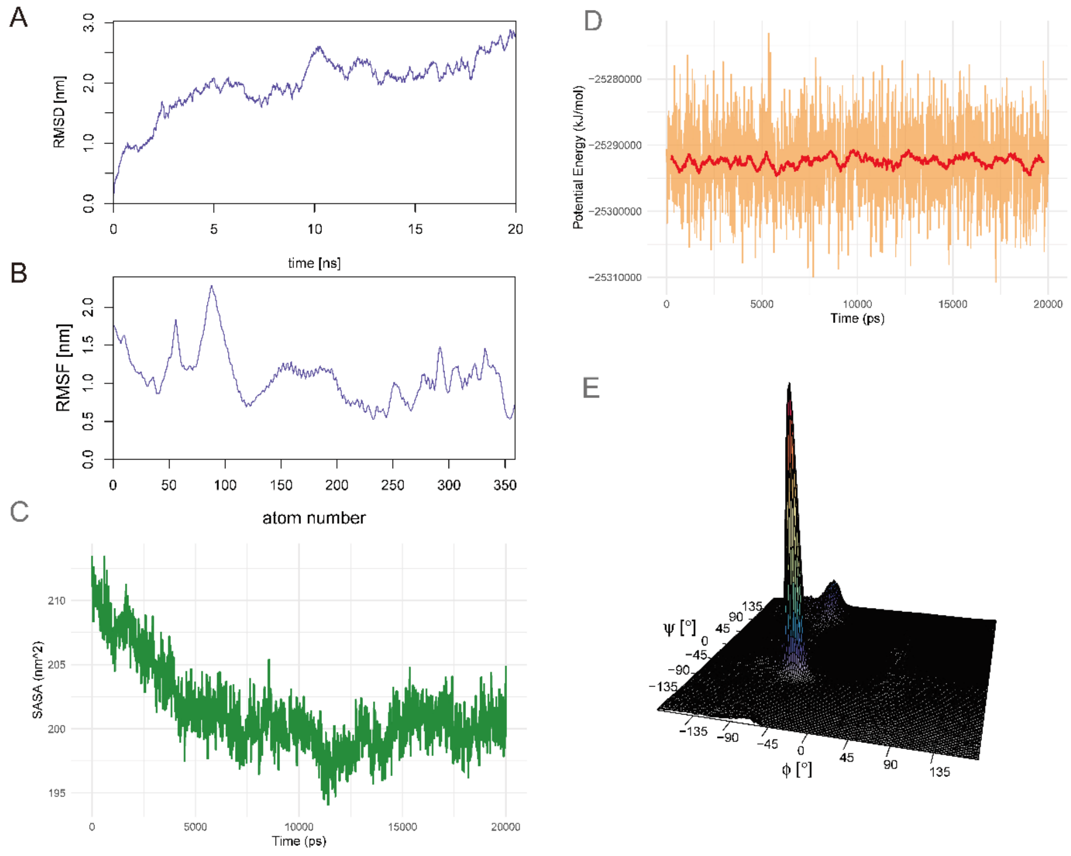

Supplementary Figure S8. Molecular dynamics (MD) simulation of CD72 in complex with dihydroergotamine.

(A) Time evolution of the root mean square deviation (RMSD) of the CD72–dihydroergotamine comple.

(B) Root mean square fluctuation (RMSF) of CD72 residues.

(C) Time evolution of the solvent accessible surface area (SASA).

(D) Potential energy profile of the system across the simulation.

(E) Ramachandran plot of CD72 backbone torsion angles.

Reference

1. Bycroft C, Freeman C, Petkova D, et al. The UK Biobank resource with deep phenotyping and genomic data. Nature 2018; 562(7726): 203-9.
2. Hemani G, Zheng J, Elsworth B, et al. The MR-Base platform supports systematic causal inference across the human phenome. Elife 2018; 7.
3. Jiang L, Zheng Z, Fang H, Yang J. A generalized linear mixed model association tool for biobank-scale data. Nat Genet 2021; 53(11): 1616-21.
4. Rashkin SR, Graff RE, Kachuri L, et al. Pan-cancer study detects genetic risk variants and shared genetic basis in two large cohorts. Nat Commun 2020; 11(1): 4423.
5. Loh PR, Kichaev G, Gazal S, Schoech AP, Price AL. Mixed-model association for biobank-scale datasets. Nat Genet 2018; 50(7): 906-8.
